# Transposition mechanism of IS*Apl1*—the determinant of colistin resistance dissemination

**DOI:** 10.1128/aac.01231-23

**Published:** 2024-01-30

**Authors:** Wei Li, Zhien He, Wei Di, Weifeng Xu, Yujie Li, Baolin Sun

**Affiliations:** 1 Department of Oncology, The First Affiliated Hospital of USTC, Division of Life Sciences and Medicine, University of Science and Technology of China, Hefei, Anhui, China; Columbia University Irving Medical Center, New York, New York, USA

**Keywords:** IS*Apl1*, Tn*6330*, *mcr-1*

## Abstract

Multidrug-resistant *Enterobacteriaceae*, a prominent family of gram-negative pathogenic bacteria, causes a wide range of severe diseases. Strains carrying the mobile colistin resistance (*mcr-1*) gene show resistance to polymyxin, the last line of defense against multidrug-resistant gram-negative bacteria. However, the transmission of *mcr-1* is not well understood. In this study, genomes of *mcr-1*-positive strains were obtained from the NCBI database, revealing their widespread distribution in China. We also showed that IS*Apl1*, a crucial factor in *mcr-1* transmission, is capable of self-transposition. Moreover, the self-cyclization of IS*Apl1* is mediated by its own encoded transposase. The electrophoretic mobility shift assay experiment validated that the transposase can bind to the inverted repeats (IRs) on both ends, facilitating the cyclization of IS*Apl1*. Through knockout or shortening of IRs at both ends of IS*Apl1*, we demonstrated that the cyclization of IS*Apl1* is dependent on the sequences of the IRs at both ends. Simultaneously, altering the ATCG content of the bases at both ends of IS*Apl1* can impact the excision rate by modifying the binding ability between IRs and ISAPL1. Finally, we showed that heat-unstable nucleoid protein (HU) can inhibit IS*Apl1* transposition by binding to the IRs and preventing ISAPL1 binding and expression. In conclusion, the regulation of IS*Apl1*-self-circling is predominantly controlled by the inverted repeat (IR) sequence and the HU protein. This molecular mechanism deepens our comprehension of *mcr-1* dissemination.

## INTRODUCTION

Recently, the misuse of antibiotics has increased multidrug-resistant bacteria, posing significant challenges for clinical treatment and prevention (1). The World Health Organization has prioritized multidrug-resistant *Enterobacteriaceae*, including *Klebsiella pneumoniae* and *Escherichia coli*, as a critical issue (2, 3). Polymyxin, especially against carbapenem-resistant *Enterobacteriaceae*, is considered the last-line defense for treating multidrug-resistant gram-negative bacteria, Despite its severe nephrotoxicity (4–6). However, bacteria have developed resistance to polymyxin, especially *via* the *mgrB* or two-component systems PmrAB, PhoPQ, and CcrAB, which target lipid A (7). In addition, in 2015, the *mcr-1* gene was reported, introducing a novel polymyxin resistance mechanism (8). Subsequently, strains positive for *mcr-1* and its variants (*mcr-2* to *mcr-10*), including *Acinetobacter baumannii*, *Salmonella enterica*, *K. pneumoniae*, and *Escherichia fergusonii*, among others, have been detected in more than 30 countries and regions worldwide (9).

Previous studies have shown that IS*Apl1* assists in the cyclization of *mcr-1* from Tn*6330* (10, 11). The IS*Apl1* gene is initially identified in *Actinobacillus pleuropneumoniae* and consists of a coding region (927 bp) flanked by an inverted repeat left (IRL), inverted repeat right (IRR) sequence (27 bp), and a direct repeat (DR) sequence (2 bp) (12). IS*Apl1* belongs to the IS*30* family and possesses the characteristic DDE domain (Asp, Asp, and Glu) found in the DD (E/D) superfamily of transposable enzymes (13, 14).

*E. coli* has the most abundant DNA binding protein heat-unstable nucleoid protein (HU), composed of two highly homologous subunits, HUα and HUβ (15). The two subunits can form as homo- or heterodimers because of the differential expression and stability of the two subunits during the growth cycle (16). It has been reported that it can regulate the transposition of Tn*10* and bacteriophage Mu (17, 18). Meanwhile, HU induces changes in gene expression by modulating the 3D arrangement of DNA. This includes altering DNA looping in the promoter region, trapping free supercoils, indirectly affecting supercoiling through DNA topoisomerases, and contacting long-range DNA-DNA interactions (19–22).

This study demonstrated the widespread distribution of *mcr-1*-positive strains in China. The cyclization of IS*Apl1*, a critical factor in regulating the propagation of *mcr-1*, is mediated by the IRs at both ends and the proteins of ISAPL1 and HU. These results offer insights into the molecular mechanisms of IS*Apl1* self-excision and the dissemination of *mcr-1*.

## RESULTS

### *mcr-1*-positive strains are distributed throughout China

**Fig 1:**
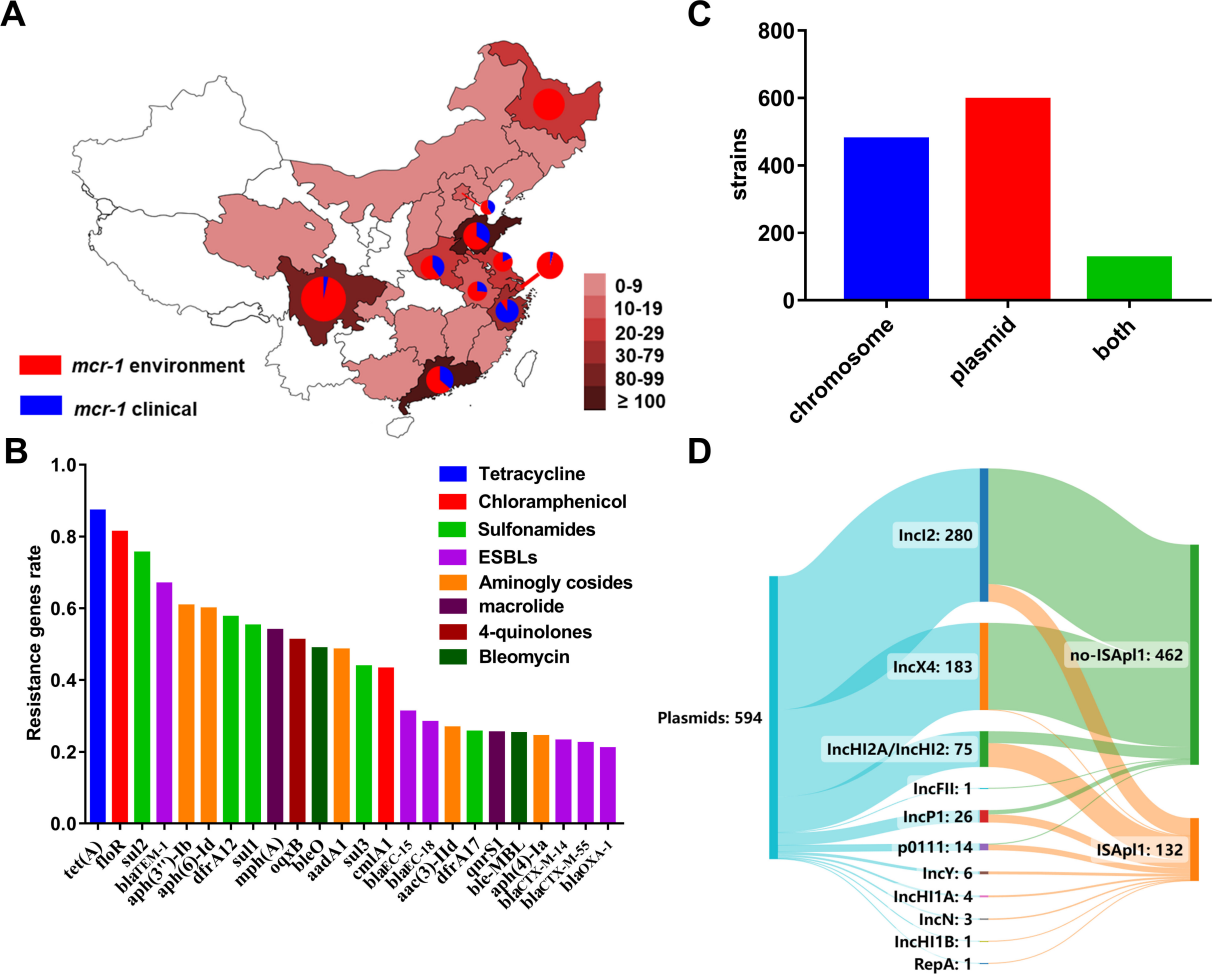
The distribution of multidrug-resistant *mcr-1*-positive strains in China. (**A**) Distribution of *mcr-1-*positive strains in China. *E. coli* genomes were downloaded from the NCBI database and statistical analysis of the regional distribution of *mcr-1*-positive strains was performed. A darker red color represents a higher number of strains. Red indicates that the strain was isolated from the environment, and blue indicates that the strain was isolated from a clinical source. (**B**) Resistance genes carried by *mcr-1*-positive strains. We used Abricate for the analysis of resistance genes. Genes resistant to the same antibiotic are marked in the same color. The proportion of resistant genes greater than 0.2 was counted. (**C**) Statistical information on the location of *mcr-1* within the genome. There are 477 samples with *mcr-1* on chromosomes (marked in blue), 594 samples with *mcr-1* on plasmids (marked in red), and 124 samples of *mcr-1* with two copies on chromosomes and plasmids (marked in green). (**D**) Types of plasmids carrying *mcr-1*. Plasmid types were categorized using a software tool named SankeyMATIC. Plasmids carrying *mcr-1* are indicated in blue, while those lacking IS*Apl1* are labeled in green, and plasmids containing IS*Apl1* are marked in orange. The thickness of the line corresponds to the number of plasmids.

To study the distribution of *mcr-1* in China. We obtained all the *E. coli* genome sequences submitted to the NCBI database from China. The research data revealed that *mcr-1*-positive strains were prevalent in various regions of China, particularly in Sichuan, Guangdong, and Shandong (Fig. 1A; supplemental Excel 1). Moreover, there was a greater occurrence of positive strains from environmental sources compared to clinical strains (Fig. 1A; supplemental Excel 1).

Previous reports have indicated that *mcr-1*-positive strains also confer resistance to other types of antibiotics (23). Consequently, we analyzed the resistance spectrum in *mcr-1*-positive strains and identified multiple antibiotic-resistant genes, including aminoglycoside, tetracycline, chloramphenicol, sulfonamide, quinolone, and β-lactam resistance-related genes (Fig. 1B; supplemental Excel 1). These findings revealed that *mcr-1*-positive isolates contained multiple drug resistance genes, implying significant challenges in clinical treatment. To gain insight into the widespread dissemination of *mcr-1*, we analyzed its location sites in the genome. The contigs of *mcr-1* were analyzed using the PlasmidFinder to ascertain its plasmid location. The findings revealed that *mcr-1* can be present in both the chromosome and plasmid (Fig. 1C), indicating its genomic transferability. The data revealed the presence of *mcr-1* in 12 distinct plasmid backgrounds, with IncI2 (47.1%) and IncX4 (30.8%) being the predominant types. Subsequently, we compared the distribution of 12 plasmid types carrying IS*Apl1*. While IncI2 and IncX4 plasmids were less prevalent, the remaining plasmid types (IncHI2A, IncP1, IncHI2, p0111, and IncY) exhibited contrasting proportions (Fig. 1D; supplemental Excel 1). The absence of IS*Apl1* may account for the stable integration of *mcr-1* in the plasmid, leading to a low carrier rate of IS*Apl1* in these plasmids. In conclusion, the study reveals that *mcr-1*-positive bacteria primarily exhibit multidrug resistance and are widely distributed across China.

### The determinant of *mcr-1* transposition element-IS*Apl1* can circle by itself

**Fig 2:**
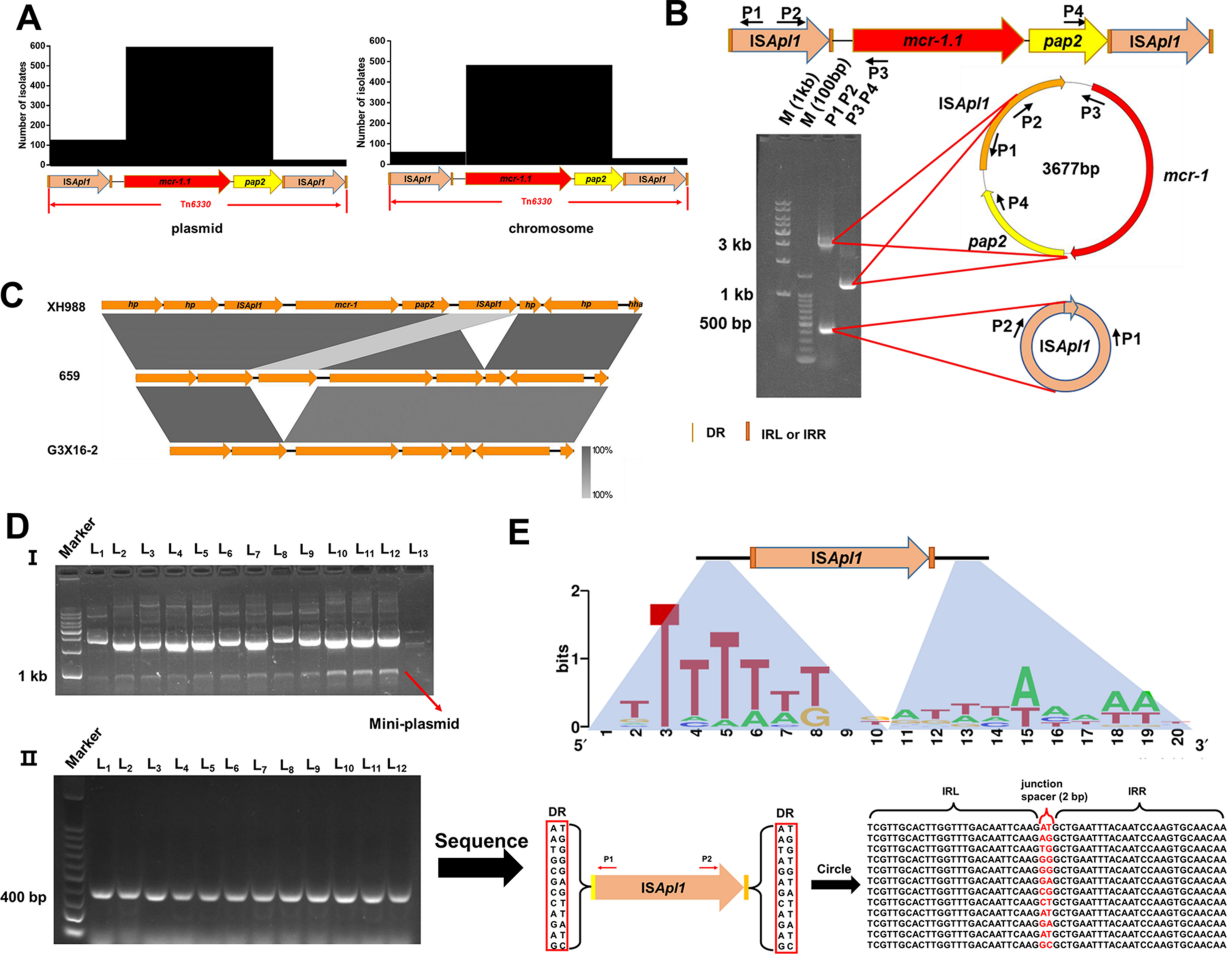
The transposition of *mcr-1* depends on IS*Apl1*. (**A**) The location of IS*Apl1* is found to be surrounding *mcr-1* in both the chromosome and plasmid. The sequences around the *mcr-1* on the plasmid and chromosome were aligned, respectively. (**B**) The excision pattern of the composite transposon Tn*6330* in 17MR471.The IS*Apl1*, *mcr-1*, and *pap2* genes are marked in orange, red, and yellow, respectively. The P1-P4 primer sequences are shown in Table S2. M: Marker. (**C**) Sequence alignment around *mcr-1* in different isolates. *mcr-1*-positive strains were compared, XH988, 659, and G3 × 16–2. The three strains sequence information from the NCBI database. (**D**) IS*Apl1* can transposition by itself. (I) We cloned IS*Apl1*, from different genome positions of 17MR471, into pUC19. Plasmids were extracted to be subjected to agarose gel electrophoresis. L_1_-L_12_ represents pUC19 containing IS*Apl1*. L_13_ represents the pUC19 wild type, serving as the negative control. (II) PCR was performed using primers P1 and P2, followed by sequencing of the PCR products. L_1_-L_12_ represents pUC19 containing IS*Apl1*, which was formed at different positions of 17MR471. Sequences in red boxes indicate DR, and bases in red font indicate junction spacer. DR: direct repeat; IRL: inverted repeat left; IRR: inverted repeat right. Red font: junction spacer. (**E**) Insertion site of IS*Apl1*. Alignment of the IS*Apl1* insertion sequence information in 17MR471 was performed using the online tool WebLogo.

To investigate the widespread distribution of *mcr-1* in China, we conducted sequence alignments surrounding the *mcr-1* gene. The findings revealed that IS*Apl1* was predominantly present upstream or downstream of the *mcr-1* gene on both plasmids and chromosomes, indicating its potential involvement in the transposition of *mcr-1* (Fig. 2A). To explore the relationship between IS*Apl1* and *mcr-1* further, we examined the environmentally isolated strain *E. coli* 17MR471, which is known to harbor Tn*6330* (24). The products generated by P1/P2 and P3/P4 primers had lengths of approximately 3 kb and 1.4 kb, respectively, suggesting that Tn*6330* was excised from *E. coli* 17MR471 in the form of IS*Apl1-mcr-1-pap2* structure (Fig. 2B). Similar findings demonstrated that the formation of a 1.4 kb product was exclusive to Tn*6330*, observed in both constructed model strain (Top10, recA^-^), as well as in the clinical strains (Fig. S1). The findings corroborated previous reports that suggested the ability of Tn*6330* to generate IS*Apl1-mcr-1-pap2* intermediates, thereby facilitating the dissemination of *mcr-1* (11). These results confirmed that the mobility of *mcr-1* relies on the intact Tn*6330* element. Through sequence alignment, we offered an initial demonstration of the evolutionary process of Tn*6330* and validated the involvement of IS*Apl1* excision and cyclization in the degradation of Tn*6330* (Fig. 2C). However, examination of the Tn*6330* cyclizing product using P1/P2 primer pair revealed the presence of an additional 400 bp product, which was subsequently sequenced and identified as IS*Apl1* self-cyclization (Fig. 2B). Whole-genome sequencing revealed 12 copies of the IS*Apl1* genes in the 17MR471. To investigate the property of IS*Apl1* self-cyclization, we inserted the IS*Apl1* at various positions in 17MR471 into pUC19 plasmids. The plasmids were subsequently extracted and analyzed using agarose gel electrophoresis. The results indicated the presence of an additional band of approximately 1 kb (mini-plasmid), known as IS*Apl1* (Fig. 2D). Following PCR amplification with P1/P2 primers, a 2 bp junction spacer, almost identical to the DR, was detected between the IRL and IRR (Fig. 2D). Subsequently, the majority of IS*Apl1* insertion sites in 17MR471 were found within the AT-rich regions (Fig. 2E). The results demonstrated that IS*Apl1* can undergo independent self-cyclization during *mcr-1* movement, thereby regulating the transmission of *mcr-1*.

### ISAPL1 transposase participates in IS*Apl1* cyclization

**Fig 3:**
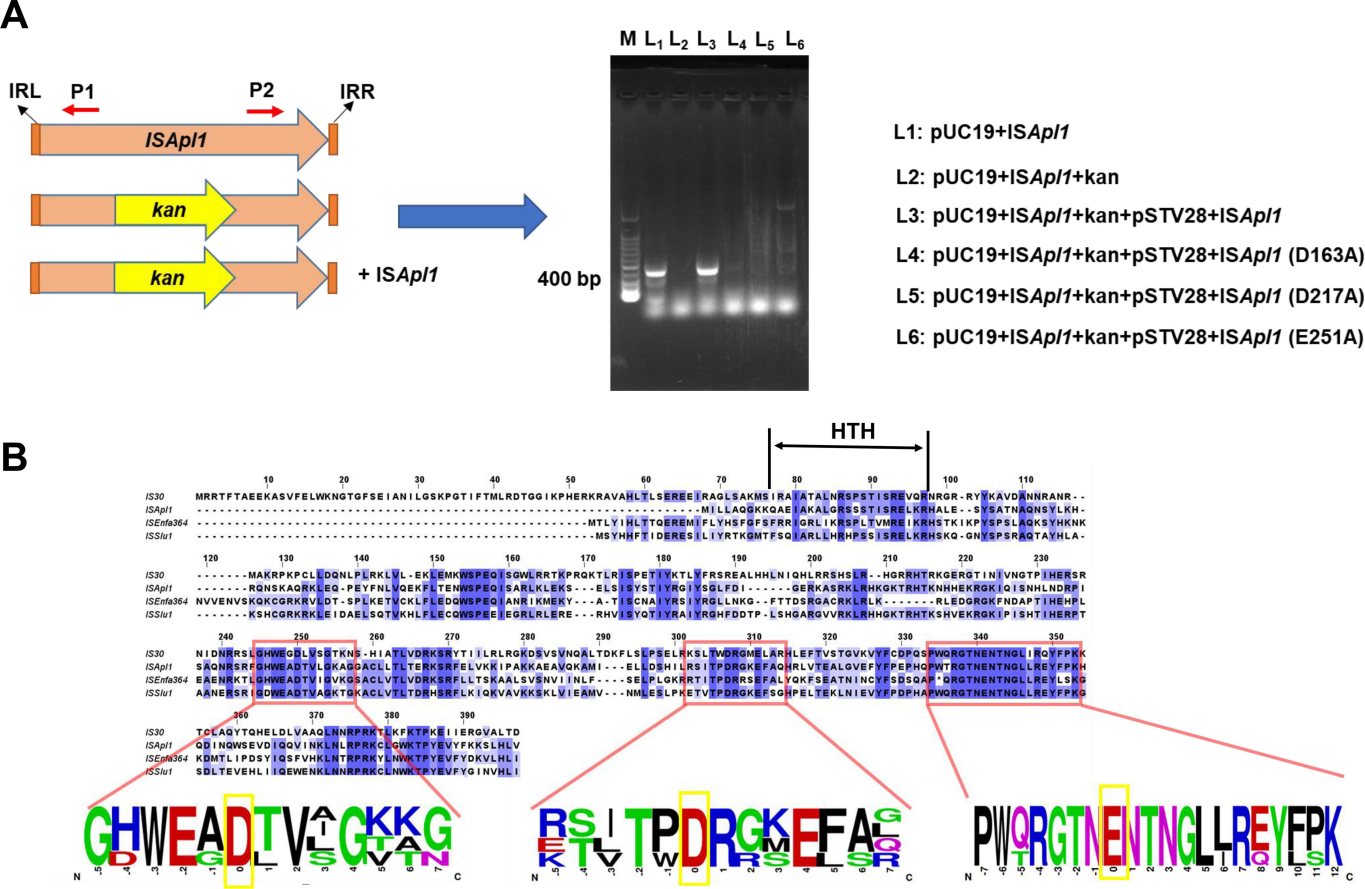
IS*Apl1* cyclization depends on the function itself. (**A**) IS*Apl1* transposition depends on the function itself. The *kan* gene was inserted into the ORF of IS*Apl1* to destroy the CDS. pSTV28 +IS*Apl1* and its point mutations D163A, D217A, and E251A plasmids were used for complement. The occurrence of transposition was verified by PCR using P1/P2 primers. The IS*Apl1* and *kan* genes are marked in orange and yellow, respectively. (**B**) IS*Apl1* transposition depends on the DDE domain. IS*Apl1* was aligned with the IS*30*, IS*Enfa364*, and IS*Slu1* sequences. The amino acid sequences around the DDE domains of the three transposases were compared. The key amino acids in the DDE domain are marked in the yellow box.

The results presented above indicate that the excision of IS*Apl1* played a role in the abortion of Tn*6330*, leading to the transposition of *mcr-1*. However, the mechanisms underlying the self-cyclization of IS*Apl1* remain unclear. Our findings demonstrated that IS*Apl1* could not transpose when disrupted by the kanamycin resistance gene (Fig. 3A). Conversely, when functional IS*Apl1* was complemented, the transposition of IS*Apl1* was detected (Fig. 3A). We conducted a preliminary exploration of the evolution of ISAPL1 through phylogenetic tree analysis, which indicated its close relation to the evolution of IS*Enfa364* and IS*Slu1*, both of which have received limited study (Fig. S2A). In addition, sequence alignment of the entire IS*30* family demonstrated high homology of ISAPL1 with other family members (Fig. S2B). To predict the function of IS*Apl1*, sequence alignment was conducted between IS*Apl1*, IS*Enfa364*, IS*Slu1*, and IS*30*. The results show that four protein sequences in the DDE (Asp, Asp, Glu)-domain were highly conserved (Fig. 3B) (12). Next, we made a single mutation of putative key sites (D163A, D217A, and E251A) on the complement IS*Apl1*. No cyclic products were observed when the mutation (D163A, D217A, and E251A) was present (Fig. 3A). These data show that IS*Apl1* cyclization was dependent on its transposase activity, especially the DDE domain.

**Fig 4:**
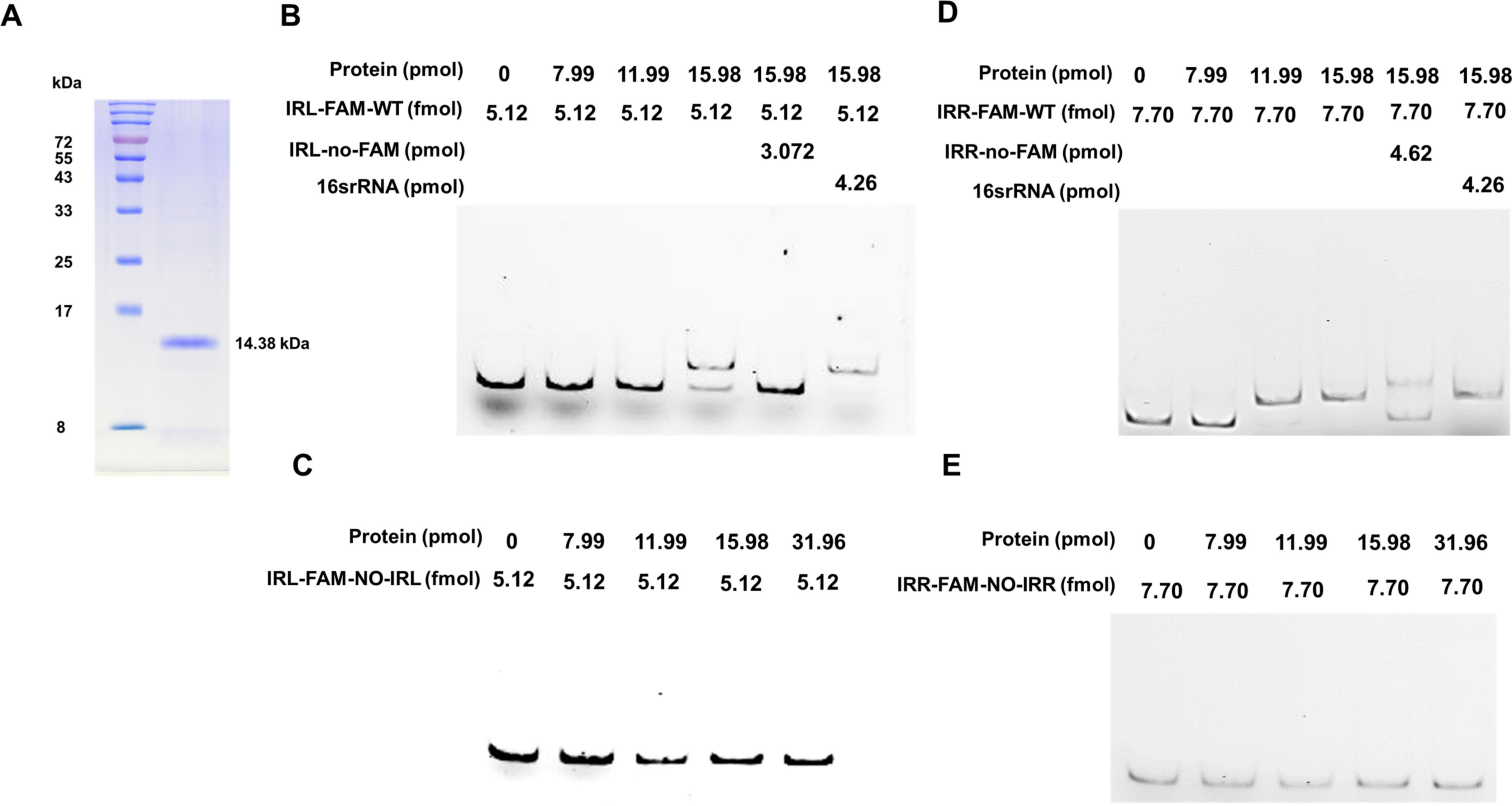
ISAPL1 binds to the sequence flanking end of IS*Apl1*. (**A**) Purification of ISAPL1 DNA-binding domain. The sequence of the IS*Apl1*-HTH domain was amplified from the 17MR471 genomic DNA. pET28a-His_6_-ISAPL1-HTH-His_6_ was used to purify the ISAPL1-HTH protein. The purified ISAPL1-HTH protein was verified by 15% SDS-PAGE followed by Coomassie blue staining. (**B**) Electrophoretic mobility shift assay—the interaction between ISAPL1 and IRL-FAM-WT. FAM-labeled probe (IRL-FAM-WT) was added to each well and incubated with the concentration gradient protein. The IRL-no-FAM probe, consistent with the IRL-FAM-WT sequence but without FAM modification, was used for a specific competition. 16SrRNA is a random sequence of DNA amplified from the genome for non-specific competition. IRL: inverted repeat left. (**C**) Electrophoretic mobility shift assay—the interaction between ISAPL1 and IRL-FAM-NO-IRL. FAM-labeled probe (IRL-FAM-NO-IRL) was added to each well and incubated with the concentration gradient protein. The probe sequence of IRL-FAM-NO-IRL means without IRL compared with IRL-FAM-WT. (**D**) Electrophoretic mobility shift assay—the interaction between ISAPL1 and IRR-FAM-WT. FAM-labeled probe (IRR-FAM-WT) was added to each well and incubated with the concentration gradient protein. The IRR-no-FAM probe without FAM modification, compared with the IRR-FAM-WT sequence, was used for a specific competition. 16SrRNA is the same to (**B**). IRR: inverted repeat right. (**E**) Electrophoretic mobility shift assay—the interaction between ISAPL1 and IRR-FAM-NO-IRR. FAM-labeled probe (IRR-FAM-NO-IRR) was added to each well and incubated with the concentration gradient protein. The probe sequence of IRR-FAM-NO-FAM means without IRR compared with IRL-FAM-WT.

To further investigate the significance of the transposition enzyme encoded by IS*Apl1* during transposition, we expressed and purified ISAPL1 protein. Unfortunately, our attempts at purification were unsuccessful. According to sequence alignment (Fig. 3B), we expressed and purified the ISAPL1-HTH (Helix Angle Helix) domain protein (Fig. 4A). An electrophoretic mobility shift assay (EMSA) experiment was performed to confirm the interaction between the ISAPL1-HTH protein and IRs of the IS*Apl1* gene. The results demonstrated the specific binding of the ISAPL1-HTH protein to IRL and IRR (Fig. 4B and D). These results uncover the function of transposase ISAPL1 which can bind the terminal sequence of IS*Apl1* and help its cyclization.

### Flanking DNA sequences at the left and right sides regulate the excision of IS*Apl1*

**Fig 5:**
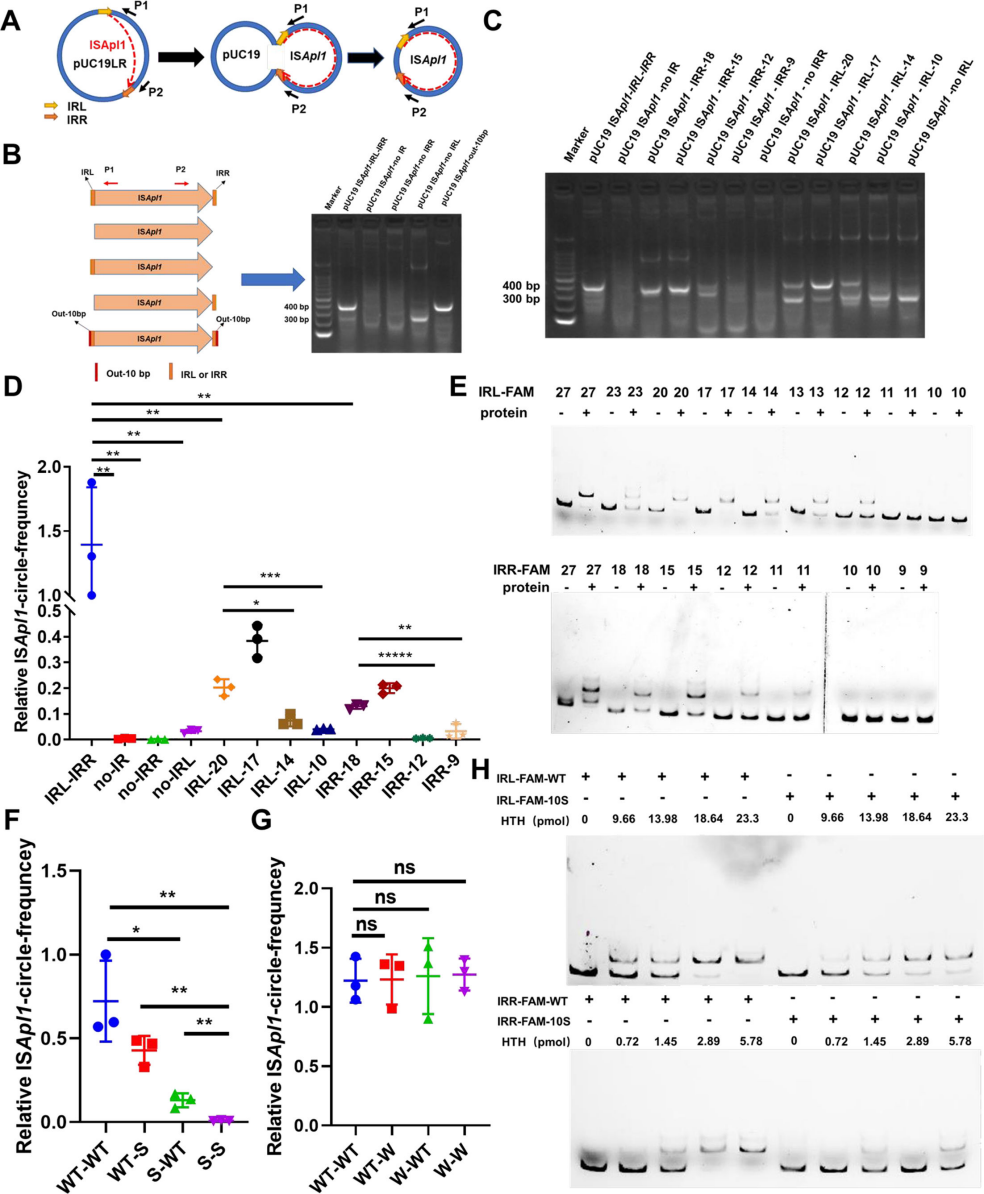
Inverted repeat (IR) sequences affect self-cyclization of IS*Apl1*. (**A**) Diagram of IS*Apl1* transposition. IS*Apl1* was cloned into Puc19 and the IS*Apl1* self-cyclization was detected using the P1/P2 primers. The product of IS*Apl1* transposition was approximately 400 bp. Yellow and orange represent IRL and IRR, respectively. (**B**) IS*Apl1* transposition is dependent on the IR at both flanking ends. Five plasmid variants—pUC19 IS*Apl1*-IRs (IRL and IRR), pUC19 IS*Apl1*-no IRs (no-IRL and no-IRR), pUC19 IS*Apl1*-no-IRR, pUC19 IS*Apl1*-no-IRL, and pUC19 IS*Apl1*-IRs-out-10 bp (contain IRL, IRR and extend of 10 base pairs outside of the IRs)—were constructed. The out-10 bp was the original sequence located in both flanking outside ends of the IR. The original sequences were “agtttaatcg” and “ggtaatattt.” The P1/P2 primer pair was used for detection. (**C**) Detection of the excision of IS*Apl1* by PCR. IRL and IRR were shortened respectively and then verified using PCR. The P1/P2 primer pair was used for detection. (**D**) RT-qPCR detection of the excision frequency of IS*Apl1* by RT-qPCR. ns, not significant; Student’s two-tailed unpaired *t*-test was utilized to calculate significant differences. *P < 0.05; **P < 0.01; ***P < 0.001; ****P < 0.0001; *****P < 0.00001. (**E**) The IRL-FAM and IRR-FAM probes were truncated and incubated with or without ISAPL1 protein. The number indicates the length of IR in the probe. “-” means no protein, “+” means with protein. (**F**) Detection of IS*Apl1* self-cyclization when the flanking bases were S (**C and G**). Primers of IS*Apl1*-circle-F and IS*Apl1*-circle-R were used to detect the frequency of IS*Apl1* self-cyclization by RT-qPCR; WT-WT (5′-agtttaatcg-IS*Apl1*-ggtaatattt-3′), WT-S (5′-agtttaatcg-IS*Apl1*-ssssssssss-3′), S-WT (5′-ssssssssss-IS*Apl1*- ggtaatattt-3′) and S-S (5′-ssssssssss-IS*Apl1*-ssssssssss-3′) (W: A, T; S: G, C). Student’s two-tailed unpaired *t*-test was utilized to calculate significant differences. *P < 0.05; **P < 0.01; ***P < 0.001; ****P < 0.0001; *****P < 0.00001. (**G**) Detection of IS*Apl1*-self-cyclization when the flanking bases were W (A, T). Primers of IS*Apl1*-circle-F and IS*Apl1*-circle-R were used to detect the frequency of IS*Apl1*-self-circle by RT-qPCR; WT-WT (5′-agtttaatcg-IS*Apl1*-ggtaatattt-3′), WT-W (5′-agtttaatcg-IS*Apl1*-wwwwwwwwww-3′), w-wt (5′-wwwwwwwwww-IS*Apl1*-ggtaatattt-3′), and w-w (5′-wwwwwwwwww-IS*Apl1*-wwwwwwwwww-3′). Student’s two-tailed unpaired *t*-test was utilized to calculate significant differences. NS, not significant (*P* > 0.05). (**H**) The ability of ISAPL1 bound to different bases bias flanking of IR. The binding ability of HTH protein with IRL-FAM-WT (6 fmol), IRR-FAM-WT (6 fmol), IRL-FAM-10S (6 fmol), IRR-FAM-10S (6 fmol) was observed by increasing the amount of protein. IRL-FAM-WT (IRL-FAM-agtttaatcg), IRR-FAM-WT (IRR-FAM-agtttaatcg), IRL-FAM-10S (IRL-FAM-SSSSSSSSSS), and IRR-FAM-10S (IRR-FAM-SSSSSSSSSS). “−” means no probe, “+” means with probe.

Despite extensive research on the functionality of the ISAPL1 transposase (Fig. 3 and 4), its excision process remains unexplored. The IS*Apl1* gene, with or without its IRL and IRR sequences, was cloned into pUC19 (Fig. 5A) and subsequently transferred into the Top10 strain (recA-) (25). Cyclization products were observed in the IRs and IRs-out-10 bp groups, whereas the no-IRs and no-IRR groups did not exhibit cyclization products (Fig. 5B). In addition, partial cyclization products of 252 bp were observed when there was no IRL (Fig. 5B). Sequencing analysis revealed that the product was attributed to homologous recombination between IRR and another CDS sequence (5′-ctcgcacagggcaaaaaacaagcagaa-3′) of IS*Apl1* (Fig. S3A). Subsequently, we conducted EMSA experiments to further validate the significance of the IR sequences. The results demonstrated that a shift was produced when the probe contained IRL (Fig. 4B), whereas no shift product was observed in the absence of IRL (Fig. 4C). The combination of IRR sequence of IS*Apl1* and ISAPL1-HTH is similar to the result of IRL (Fig. 4D and E).

To investigate the impact of IR length on IS*Apl1* excision, we shortened the original IRL and IRR sequences. The findings revealed that the minimum length of IS*Apl1* transposition-dependent IRR was 12 bp (Fig. 5C), and a mismatch occurred when the IRR was truncated to 18–15 bp (Fig. S3C). Surprisingly, IS*Apl1* could transpose in a mismatched manner when there was NO-IRL (Fig. S3B). To achieve transposition without mismatches, the minimum length of IRL was 14 bp (Fig. 5C). In addition, the RT-qPCR revealed that the excision rate of IS*Apl1* corresponded to the length of the IRs (Fig. 5D). Furthermore, the EMSA experiment showed that the binding ability of the probes with the protein decreased with the shortening of IRL and IRR length (Fig. 5E). These findings provided further confirmation that the IRs were essential for the transposition of IS*Apl1*.

In addition, the excision frequency was found to be significantly lower in the pUC19-IS*Apl1*-IRs group (“5′-aagcttgtga-IS*Apl1*-gtcgactcta-3′”) compared to the pUC19-IS*Apl1*-IRs-out-10-bp group (5′-agtttaatcg-IS*Apl1*-ggtaatattt-3′) (Fig. S4). It is interesting to note that the pUC19-IS*Apl1*-IRs-out-10-bp group had a higher AT content in the flanking end of IRs. Subsequently, 10 bp random sequences S (C, G) or W (A, T) were added to the outside region of IRL and IRR to construct pUC19 variants. The excision rate of IS*Apl1* significantly decreased when it was flanked by CG-rich sequences (Fig. 5F). However, the rate was no significantly different when it was flanked by AT-rich sequences (Fig. 5G). Subsequently, we conducted EMSA experiments to determine whether the excision rate decreased due to the binding ability of ISAPL1 and the IRs at both ends. The results revealed that IRL-FAM-WT and IRR-FAM-WT had stronger binding abilities to ISAPL1 compared to IRL-FAM-10S and IRR-FAM-10S (Fig. 5H). As illustrated in Fig. 2E, the IS*Apl1* insertion site was primarily located in regions with high AT regions, which conferred its excision activity. In conclusion, the excision of IS*Apl1* was affected by the length of the IRs and the base bias at both flanking ends, which primarily affected the binding ability with ISAPL1.

### *hupA* or *hupB* can regulate IS*Apl1*-self excision

**Fig 6:**
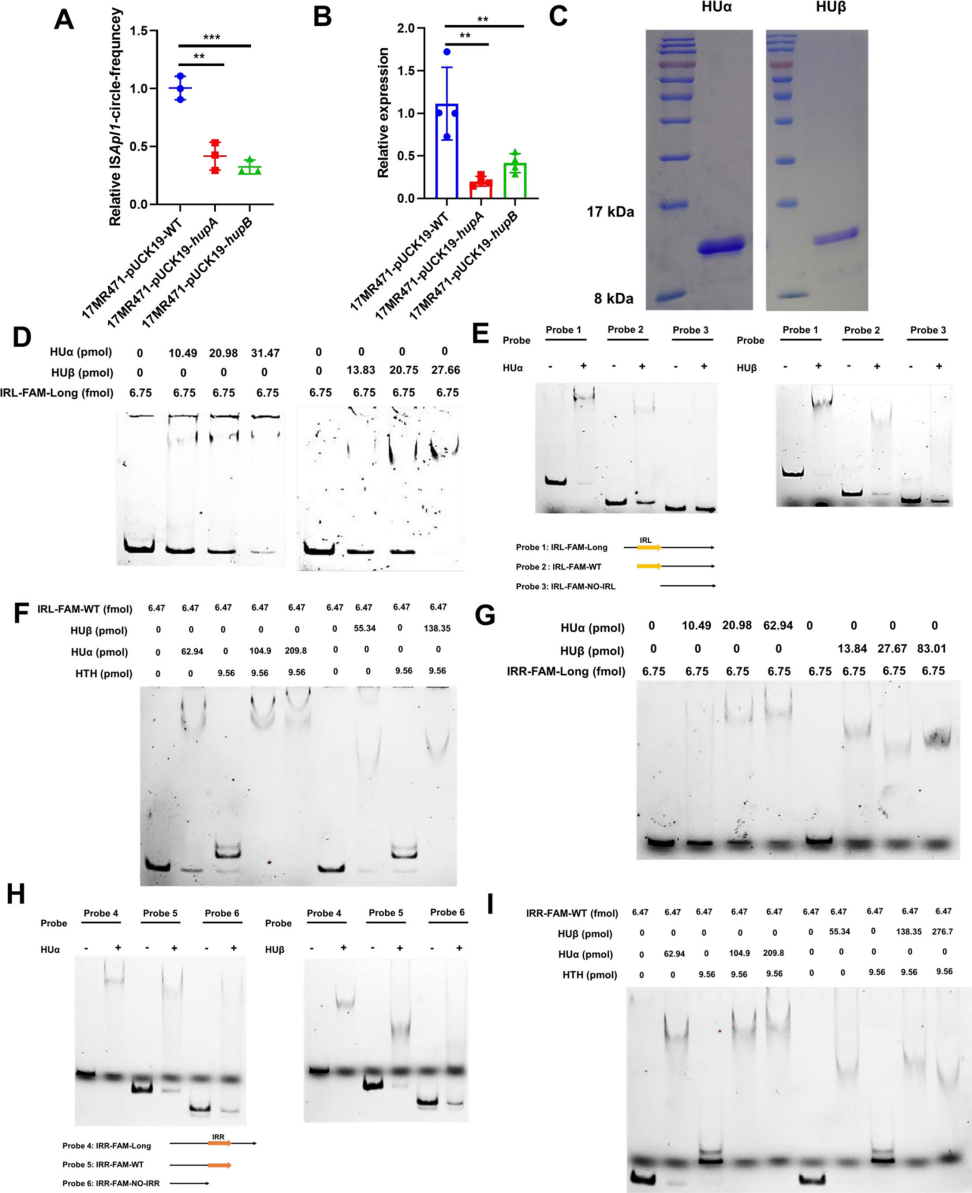
HU can inhibit the excision of IS*Apl1* (**A**) Overexpression of *hupA* or *hupB* in 17MR471 was followed by whole-genome extraction to examine the excision of IS*Apl1*. 16SrRNA was used as an internal control. Student’s two-tailed unpaired *t*-test was utilized to calculate significant differences. *P < 0.05; **P < 0.01; ***P < 0.001. (**B**) Overexpression of *hupA* or *hupB* in 17MR471 was followed by RNA extraction and detection of IS*Apl1* expression. 16SrRNA was used as an internal control. Student’s two-tailed unpaired *t*-test was utilized to calculate significant differences. *P < 0.05; **P < 0.01; ***P < 0.001. (**C**) Expression and purification of HUα and HUβ proteins. (**D**) The promoter of IS*Apl1* (containing IRL) was incubated with HUα or HUβ proteins, respectively. IRL-FAM-Long was amplified using primers IS*Apl1*-R-FAM, IS*Apl1*-F-probe. (**E**) IRL-FAM-long probes were truncated and incubated with HUα or HUβ proteins. Probe 1: IRL-FAM-Long (IS*Apl1*-R-FAM, IS*Apl1*-F-Probe); Probe 2: IRL-FAM-WT (IS*Apl1*-R-FAM, IS*Apl1*-F-IRL-27); Probe 3 (IS*Apl1*-R-FAM, IS*Apl1*-F-NO-IRL). Probe 1 and Probe 2 with the IRL, Probe 3 without IRL. IRL was presented with a yellow color. (**F**) HUα or HUβ competed with ISAPL1 for IRL. The HUα: ISAPL1, HUΒ: ISAPL1 hybrid proteins were incubated with the probe (IRL-FAM-WT), and the HUα or HUβ content was gradually increased. (**G**) The IRR sequence of IS*Apl1* was incubated with HUα and HUβ proteins, respectively. IRR-FAM-Long was amplified using primers IS*Apl1*-F-FAM, *mcr-1*-R-probe. (**H**) IRR-FAM-long probes were truncated and incubated with HUα or HUβ proteins. Probe 4: IRR-FAM-Long (IS*Apl1*-F-FAM, *mcr-1*-R-probe); Probe 5: IRR-FAM-WT (IS*Apl1*-F-FAM, IS*Apl1*-R-IRR-27); and Probe 6: IRR-FAM-NO-IRL (IS*Apl1*-F-FAM, IS*Apl1*-R-NO-IRR). IRR sequences are shown in orange. (**I**) HUα or HUβ competed with ISAPL1 for IRR. The HUα: ISAPL1, HUβ: ISAPL1 hybrid proteins were incubated with the probe (IRR-FAM-WT), and the HUα or HUβ content was gradually increased.

To further explore the novel mechanism of IS*Apl1* excision regulation, a pull-down assay was performed, and the results were analyzed by SDS-PAGE and mass spectrum (Fig. S5; Excel 2 and 3). HUα and HUβ, which are encoded by *hupA* and *hupB* respectively, were chosen as our target proteins (Table 1). Heat-unstable nucleoid protein (HU) has been reported to be involved in the global regulation and mediation of transposition (26, 27). To explore the role of *hupA* and *hupB* in IS*Apl1* transposition, we overexpressed the two genes and detected the excision rate in 17MR471. The results showed that the excision rate of IS*Apl1* was suppressed by *hupA* or *hupB* (Fig. 6A). In addition, the expression of IS*Apl1* was evaluated, revealing that *hupA* or *hupB* could suppress the expression of IS*Apl1*, thereby regulating its excision (Fig. 6B). We failed to knock out *hupA* or *hupB* in 17MR471 due to its multi-resistance. Consequently, we knocked out *hupA* or *hupB* in *E. coli* Top10 and transformed the mutational strain with pUC19 + WT-IS*Apl1*-WT. Unfortunately, the deletion of *hupA* or *hupB* did not affect the excision of IS*Apl1* (Fig. S6). To further explore the mechanism of *hupA* or *hupB* regulated the expression of IS*Apl1*, HUα, and HUβ proteins were purified (Fig. 6C). EMSA assay confirmed their binding to the IS*Apl1* promoter (Fig. 6D). Therefore, HUα or HUβ suppressed the expression of IS*Apl1* by directly binding to its promoter. To investigate the specific binding sites of HUα and HUβ to the IS*Apl1* promoter, the IRL-FAM-long probe was shortened. The results showed that HUα or HUβ could bind to IRL-FAM-long or IRL-FAM-WT probes (with IRL sequences) but no shift was observed when IRL-FAM-NO-IRL was employed (without IRL sequences) (Fig. 6E). This suggests the binding site of HUα and HUβ is the IRL sequence. However, the HTH domain of ISAPL1 also binds to the IR sequence (Fig. 4). We hypothesized that HUα and HUβ regulated the transposition of IS*Apl1* by competing with ISAPL1 transposase for the same DNA sequence. Subsequently, we conducted competition experiments. The results showed that HUα or HUβ can displace ISAPL1 from the IRL-FAM-WT sequence (Fig. 6F). HUα and HUβ bound to the IRL sequence and regulated the excision of IS*Apl1* suggests that they may also bind to the IRR sequence, impacting the regulation of ISAPL1 transposition. To investigate this, an EMSA was performed using HUα or HUβ proteins incubated with the IRR-FAM-long probe. The experiment results revealed that both HUα and HUβ could bind to the probe (Fig. 6G). In addition, we observed that truncating IRR-FAM-long resulted in a notable decrease in the binding ability of IRR-FAM-NO-IRR (lacking the IRR sequence) when compared to IRRL-FAM-Long (with IRR) and IRR-FAM-WT (with IRR) (Fig. 6H). Furthermore, competition experiments demonstrated that both HUα or HUβ could bind to the IRR sequence, inhibiting ISAPL1 binding to IRR (Fig. 6I). The above results show that HUα or HUβ inhibit ISAPL1 transposition through two aspects: suppressing IS*Apl1* expression and competing for the same DNA sequence as ISAPL1.

**TABLE 1:** Proteins from Pull-down

Protein	Function^*a*^
HUα Uniprot: A0A0E0VE47	Histone-like DNA-binding protein which is capable of wrapping DNA to stabilize it
HUβ Uniprot: A0A0A5SD62	Histone-like DNA-binding protein which is capable of wrapping DNA to stabilize it

^*a*^ The database from Uniport.

### IS*Apl1* can relieve the inhibition of HU

**Fig 7:**
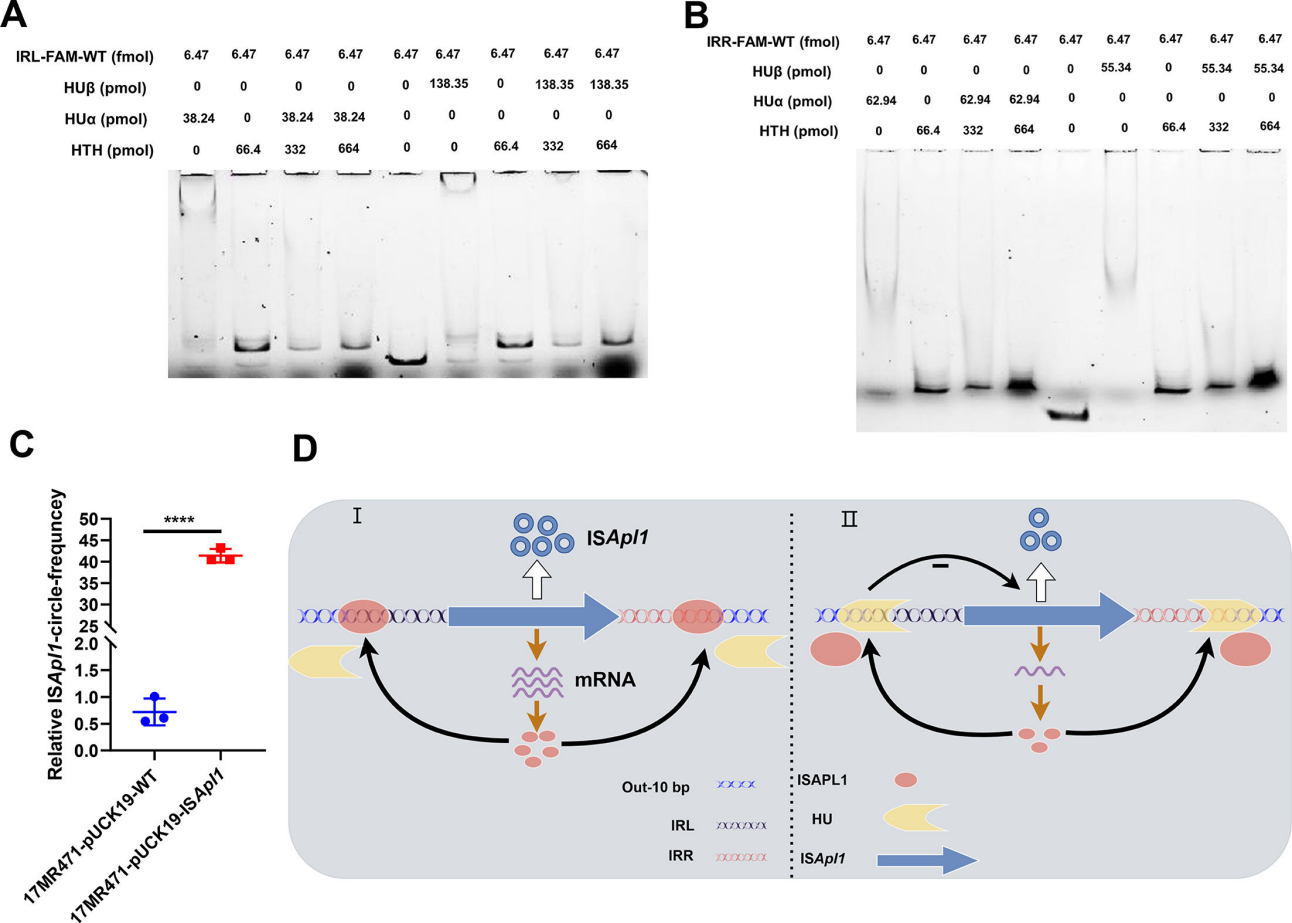
ISAPL1 can restore the excision of IS*Apl1*. (**A**) ISAPL1 competed with HUα or HUβ for IRL, respectively. The ISAPL1: HUα, ISAPL1: HUβ mixture proteins were incubated with a probe (IRL-FAM-WT), and the content of ISAPL1 was gradually increased. (**B**) ISAPL1 competed with HUα or HUβ for IRR, respectively. ISAPL1: HUα, ISAPL1: HUβ mixture proteins were incubated with a probe (IRR-FAM-WT) and the content of ISAPL1 was gradually increased. (**C**) IS*Apl1* can promote the excision of IS*Apl1.* We overexpressed IS*Apl1* in 17MR471 and detected the excision of IS*Apl1*.16SrRNA as an internal reference. Student’s two-tailed unpaired *t*-test was utilized to calculate significant differences. *P < 0.05; **P < 0.01; ***P < 0.001; ****P < 0.0001. (**D**) Schematic of HU-regulated IS*Apl1* transposition. (I) ISAPL1 transposase can bind to IRs, and its binding ability is affected by the length of IRs and the base bias on the flanking of IRs. (II) HU could bind to IRs to inhibit the expression of IS*Apl1* and compete with ISAPL1 transposase for the same DNA sequence.

The interaction between HU and IRL or IRR sequences inhibits the transposition of IS*Apl1* by competitively binding to the same DNA sequence as ISAPL1 (Fig. 6). To determine whether the inhibitory effect of HU on IS*Apl1* transposition could be reversed by increasing the amount of ISAPL1, a competition experiment was conducted. HUα, HUβ, or ISAPL1 proteins were separately incubated with IRL-FAM-WT probe. Subsequently, ISAPL1 was incubated with HUα or HUβ, and the ISAPL1:HUα and ISAPL1:HUβ ratios were gradually increased. The results showed that ISAPL1: HUα generated the same shift as ISAPL1 instead of HUα or HUβ (Fig. 7A). Similar outcomes were observed with IRR-FAM-WT probe (Fig. 7B), indicating that ISAPL1 can displace HU from the IRL or IRR sequences, thereby restoring IS*Apl1* transposition. To validate this, IS*Apl1* was overexpressed and examined for transposition. The results demonstrated a significantly higher transposition frequency of IS*Apl1* compared to the WT, *hupA*, and *hupB* groups (Fig. 6A and 7C). Overall, these findings suggest that overexpression of IS*Apl1* relieves the inhibitory effect of HU on IS*Apl1* transposition.

## DISCUSSION

Molecular epidemiology and retrospective studies have shown that food animals may serve as the origin of *mcr-1*-positive isolates (28). The plasmid pHNSHP45 (contained *mcr-1*) is detected from SHP45 isolate, which is separated from animal, was detectable in various bacteria, and can be transmitted (8). Subsequently, a variant of pHNSHP45, known as *mcr-1*-carrying conjugative plasmid, was found to have spread among *Salmonella* isolates obtained from humans, pigs, and chickens (29). The transmission of the *mcr-1* gene on the plasmid may be the key factor in its transmission from animals to humans. This could explain why the number of environmental isolates exceeds that of clinical isolates (Fig. 1A). Moreover, the variation in the regional distribution of strains in China may also be influenced by several factors, including economic development, the number of hospitals and scientific research institutions, and research capacity.

To date, a total of 15 Inc-type plasmids carrying *mcr-1* have been documented (30). Among these, IncX4, IncHI2, and IncI2 have emerged as the predominant plasmids carrying *mcr-1* (9, 31). Our findings reveal the presence of 12 types of plasmids carrying *mcr-1*, with IncI2 and IncX4 being the primary types responsible for the transmission of *mcr-1*. In addition, we observed a higher frequency of plasmids carrying IS*Apl1* when compared to the predominant plasmids (IncI2, IncX4). These other plasmids (IncHI2A, IncP1, IncHI2, P0111, IncY, IncHI1A, IncN, IncHI1B, IncFII, RepA) exhibited a high frequency of carrying IS*Apl1*. The presence of both IS*Apl1* and *mcr-1* in a plasmid may confer the ability of *mcr-1* transposition, which could explain the higher frequency of *mcr-1* in IncI2- and IncX4-type plasmids.

The occurrence of transposition can be influenced by various factors, including transcription factors (26), the sequence at the ends of transposons (32–34), and the regulation of transposases (25, 35). Our results show that multiple factors affect IS*Apl1* transposition. These factors include the IRs and the base bias at both the flanking ends of IS*Apl1*, as well as the transposase encoded by IS*Apl1* and the HU protein. Previous reports have highlighted the significance of transcription factors, such as IHF, FIS, and H-NS, in regulating transposition by binding to DNA sequences of transposons (32, 36–38). For instance, H-NS has been shown to bind to the terminal sequence of Tn*5* transposons, thus modulating their transposition (32). IHF and H-NS have also been implicated in mediating the dissociation of a refractory protein-DNA composite during Tn*10*/IS*10* transposition (39). Our results indicate a significant decrease in the excision rate of IS*Apl1* when *hupA* or *hupB* is overexpressed. Further research is needed to determine whether the effect is exerted by the homodimer (HUαα, HUββ) or the heterodimer (HUαβ). In addition, when we knocked out *hupA* or *hupB* in the TOP10 strain, there was no change in IS*Apl1* transposition. This suggests that the regulation of IS*Apl1* transposition by HU may be strain-specific.

The transfer of *mcr-1* relies on the Tn*6330* complex transposon but the molecular mechanism of how IS*Apl1* facilitates *mcr-1* transmission requires further investigation. Erik Snesrud et al. have demonstrated that the birth and demise of the IS*Apl1-mcr-1*-IS*Apl1* composite transposon hinged on the specific recognition and insertion of the *mcr-1* surrounding sequence by IS*Apl1*, as determined through comparative genomics (40). Furthermore, the stability of *mcr-1* in the genome is contingent upon the deletion of both ends of the IS*Apl1* sequence (9). More experiments may be needed to verify the relationship between IS*Apl1* and *mcr-1*. The presence of IS*Apl1* surrounding *mcr-1* in *mcr-1*-positive strains has been frequently observed (9, 41, 42) but the proportion of IS*Apl1* in relation to *mcr-1* has yet to receive much attention. We evaluated the probability of IS*Apl1* quantitatively surrounding *mcr-1*. Out of the 947 *mcr-1*-positive strains, 66 were identified through third-generation sequencing, while 881 were identified through second-generation sequencing. It is important to note that the limitations of second-generation sequencing technology may influence this approximate result. Through sequence comparison, we discovered that the presence of IS*Apl1* upstream and downstream of *mcr-1* accounted for 17.6% and 5.2%, respectively. Whenever IS*Apl1* is detected downstream of *mcr-1*, it is always accompanied by IS*Apl1* upstream; in other words, the structure of *mcr-1-pap2-*IS*Apl1* has yet to be found to exist. The neighboring sequences of IS*Apl1* potentially influence this observation. Previous studies have demonstrated that the DRs on both sides of IS*Apl1* remain consistent when inserted into a novel location (43). However, when comparing the sequence at both ends of IS*Apl1* around *mcr-1*, we found that the DRs on both sides upstream and downstream of IS*Apl1* are inconsistent but in the structure of Tn*6330,* the DRs of its were consistent. Wang et al. showed that the Tn*6330* element synchronizes the DRs at both flanking ends by transposing them to a new position (9).

In this study, we discovered the widespread distribution of *mcr-1*-positive bacteria across China. We also found that IS*Apl1*, a crucial factor in *mcr-1* transmission, is capable of self-transposition. In addition, the self-cyclization of IS*Apl1* relied on the involvement of its encoded transposase. Moreover, the excision of IS*Apl1* was influenced by the length of IRs and the nucleotide bias at both flanking ends. Importantly, the excision of IS*Apl1* was influenced by HU, which suppressed the expression of IS*Apl1* and competed for the same DNA with ISAPL1. These results give us a better understanding of the molecular mechanism of *mcr-1* dissemination.

## MATERIALS AND METHODS

### Bacterial strains, plasmids, and growth conditions

The strains and plasmids are listed in Table S1. *E. coli* cultures were grown in a Luria broth medium (Oxoid). Plasmid maintenance involved antibiotic concentrations of chloramphenicol (15 µg/mL), ampicillin (150 µg/mL), and kanamycin (50 µg/mL). In addition, we obtained 2,463 whole-genome sequences of *E. coli* from the NCBI database (https://www.ncbi.nlm.nih.gov/pathogens/isolates/). These strains were extensively distributed across 33 regions of China, encompassing both environmental and clinical isolates.

### Analysis of genome profiling and comparative genomics

ABRicate (44) was employed to identify resistance genes and plasmids in *mcr-1*-positive isolates. The results, aligned using Clustal Omega (45–47), were manually edited and corrected using Jalview. WebLogo (48) was used to align the surrounding sequence of IS*Apl1* insertion sites. The IS*30* family protein sequence was downloaded from the ISfinder database. MAGE was used to generate a phylogenetic tree, which was edited and enhanced using iTOL (https://itol.embl.de/upload.cgi).

### Plasmid construction

A series of plasmids derived from pUC19, including IR truncation, base preference at both flanking ends of IR, were constructed. The primers used for constructing are listed in Table S2. To generate IS*Apl1* mutant strain, we inserted the kanamycin resistance gene sequence into IS*Apl1*. In addition, we created plasmids pSTV28 + IS*Apl1* and its point mutations (D163A, D217A, E251A) for complementation. To examine the impact of *hupA* and *hupB* on excision, we individually ligated them to pUCK19 plasmid (49).

### Expression and purification of ISAPL1-HTH domain, HUα, and HUβ proteins

*E. coli* BL21 cells that carried the respective plasmids were incubated in LB at 37°C to an OD_600_ of 0.5 and induced with 1 mM IPTG at 16°C for 20 h. The His-tagged proteins were purified using Purification Kit (P2226, Beyotime) and confirmed by 15% SDS-PAGE followed by Coomassie blue staining, and their concentrations were determined using Bradford Protein Assay Kit (P0006C, Beyotime).

### Electrophoretic mobility shift assay

Fluorescein amidite (FAM)-labeled probes were incubated with proteins in binding buffer (50 mM NaH_2_PO_4_, 300 mM NaCl, 50 mM imidazole) at 25°C for 30 min. After incubation, a 5% native polyacrylamide gel in 1× Tris-borate-EDTA (TBE) buffer was used, applied at 140 V for 30 min at 4°C. The bind shifts were detected by Amersham Typhoon NIR (GE) and analyzed by ImageQuant TL 8.1.

### Knockout *hupA* and *hupB*

The *hupA* or *hupB* knockout strains were generated using red recombination. Briefly, the pKD46 plasmid was transformed into the wild-type (WT) strain. Then, the chloramphenicol resistance (*cat*) gene, flanking by 40 bp homology arms located upstream and downstream of the target gene, was amplified *via* PCR using pKD3 as a template. Then, the cells were induced by L-arabinose. The positive strain was cultured in LB without antibiotics at 42°C for 16 hours to remove pKD46. The pCP20 plasmid was used to remove the *cat* gene. To eliminate the pCP20, the overnight culture was incubated at 30°C and then subjected to a 48-hour incubation at 42°C.

### RT-qPCR detection of excision frequency

Plasmids or genomes were extracted and the excision rate of IS*Apl1* was determined using RT-qPCR. 16SrRNA served as the internal reference.

### Total RNA extraction and RT-qPCR

An overnight culture of 17MR471 was prepared. The bacterial cells were collected by centrifugation at 12,000 × *g* for 1 minute. Next, 1 mL of RNAiso plus (108–95-2, TaKaRa) was added. The cells were then disrupted using 0.1 mm silica beads and a fast PRE24 automated system (MP Biomedicals). RNA was reverse-transcribed into cDNA using the PrimeScript RT reagent kit (RR420A, TaKaRa), and 16SrRNA served as the internal reference.

### DNA Pull-down assay

DNA pull-down assays were conducted as previously described (50). The biotin-labeled IS*Apl1* promoter was amplified from the genomic DNA of 17MR471. 16SrRNA-biotin was used as a negative control. Cell cultures were inoculated into 20 mL of fresh LB medium and incubated overnight. The cells were then collected and lysed. The lysate was then centrifuged at 12,000 × *g* at 4°C for 40 minutes to remove insoluble debris. The supernatant containing 10 µg/mL of poly (dI-dC) was added to the DNA-coated beads and incubated at 4°C for 1 hour. Then, the beads were supplemented with ddH_2_O (70 µL) and incubated at 70°C for 10 minutes. Samples were separated by SDS-PAGE. The entire lanes containing IS*Apl1* and 16SrRNA were excised and subjected to in-gel digestion with trypsin (0.6 mg). The resulting tryptic peptides were analyzed using liquid chromatography-tandem mass spectrometry (LC-MS/MS) with an LTQ mass spectrometer (ProteomeX-LTQ; ThermoFisher Scientific). Sequence and peptide fingerprint data were then analyzed using the NCBI database.

### Statistical analysis

All experiments were performed in biological triplicates. GraphPad Prism (version 8.3d) was employed for all statistical analyses. Student’s two-tailed unpaired *t*-test was utilized to calculate significant differences. NS, not significant (*P* > 0.05); *P < 0.05; **P < 0.01; ***P < 0.001; ****P < 0.0001.

## SUPPLEMENTAL MATERIAL

Supplemental Excel 1 aac.01231-23-s0001.xlsx List of *mcr-1*-positive isolates.

Supplemental Excel 2 aac.01231-23-s0002.xlsx Pull-down for IS*Apl1*.

Supplemental Excel 3 aac.01231-23-s0003.xlsx Pull-down for 16S rRNA.

Supplemental Fig S1 aac.01231-23-s0004.tif the cyclization of *Tn6330*.

Supplemental Fig S2 aac.01231-23-s0005.tif IS*30* family evolutionary tree and IS*30*-family-align.

Supplemental Fig S3 aac.01231-23-s0006.tif Sanger sequence of cyclization product.

Supplemental Fig S4 aac.01231-23-s0007.tif Detection of cyclization frequency.

Supplemental Fig S5 aac.01231-23-s0008.tif Pull-down.

Supplemental Fig S6 aac.01231-23-s0009.tif Detection of cyclization frequency.

Supplemental Fig. S1-S7 and Tables S1-S2 aac.01231-23-s0010.docx Fig. S1-S7 and Excel S1-S3 descriptions and lists of isolates, plasmids, and primers.

ASM does not own the copyrights to Supplemental Material that may be linked to, or accessed through, an article. The authors have granted ASM a non-exclusive, world-wide license to publish the Supplemental Material files. Please contact the corresponding author directly for reuse.
