# Supplementary figures and images for: Transposition mechanism of ISApl1—the determinant of colistin resistance dissemination
Source: Antimicrob Agents Chemother. 2024 Jan 30;68(3):e01231-23. doi: 10.1128/aac.01231-23 (PMC10916398; doi:10.1128/aac.01231-23)

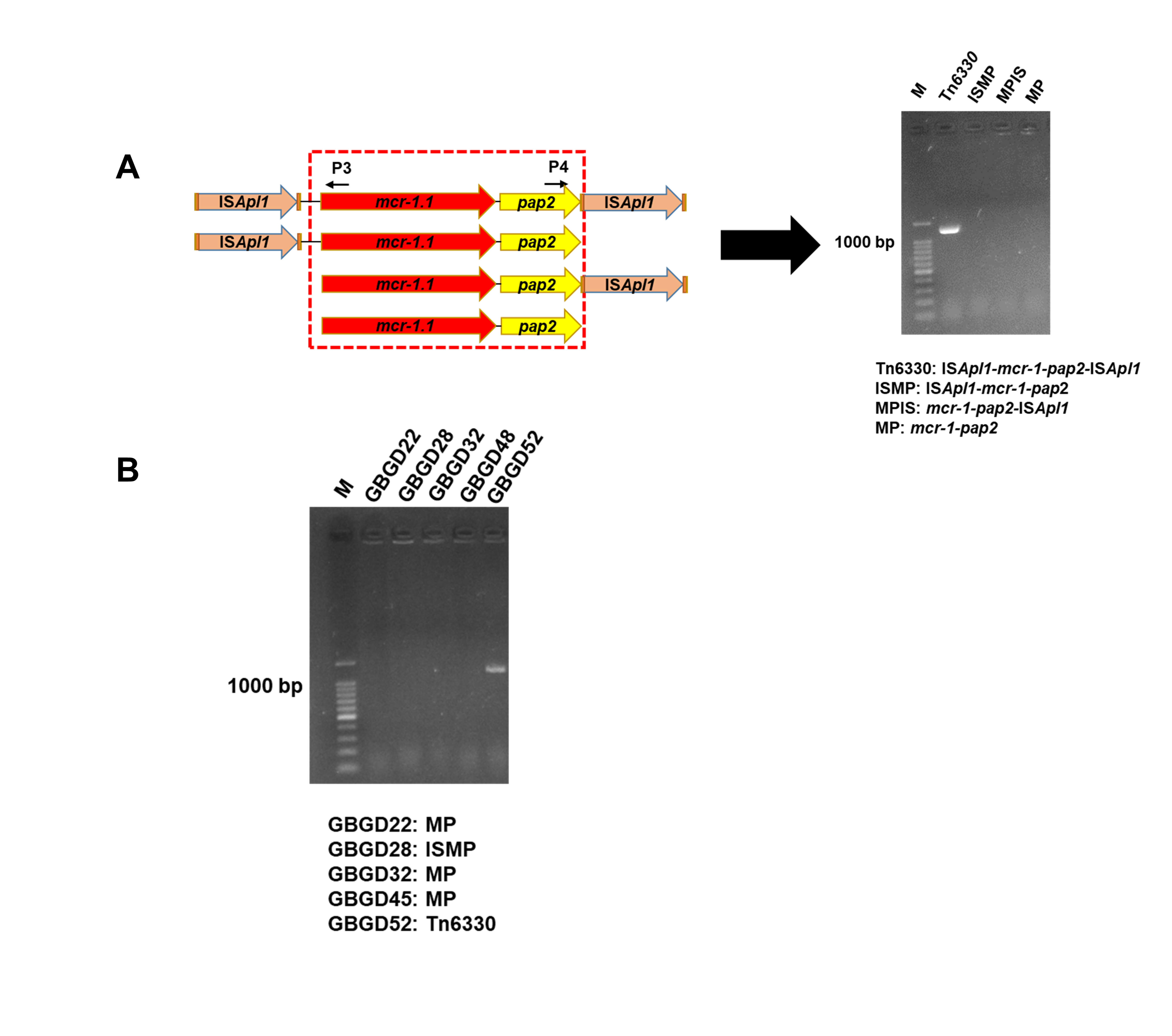

Supplement: Supplemental Fig S1 — the cyclization of Tn6330. [file aac.01231-23-s0004.tif]

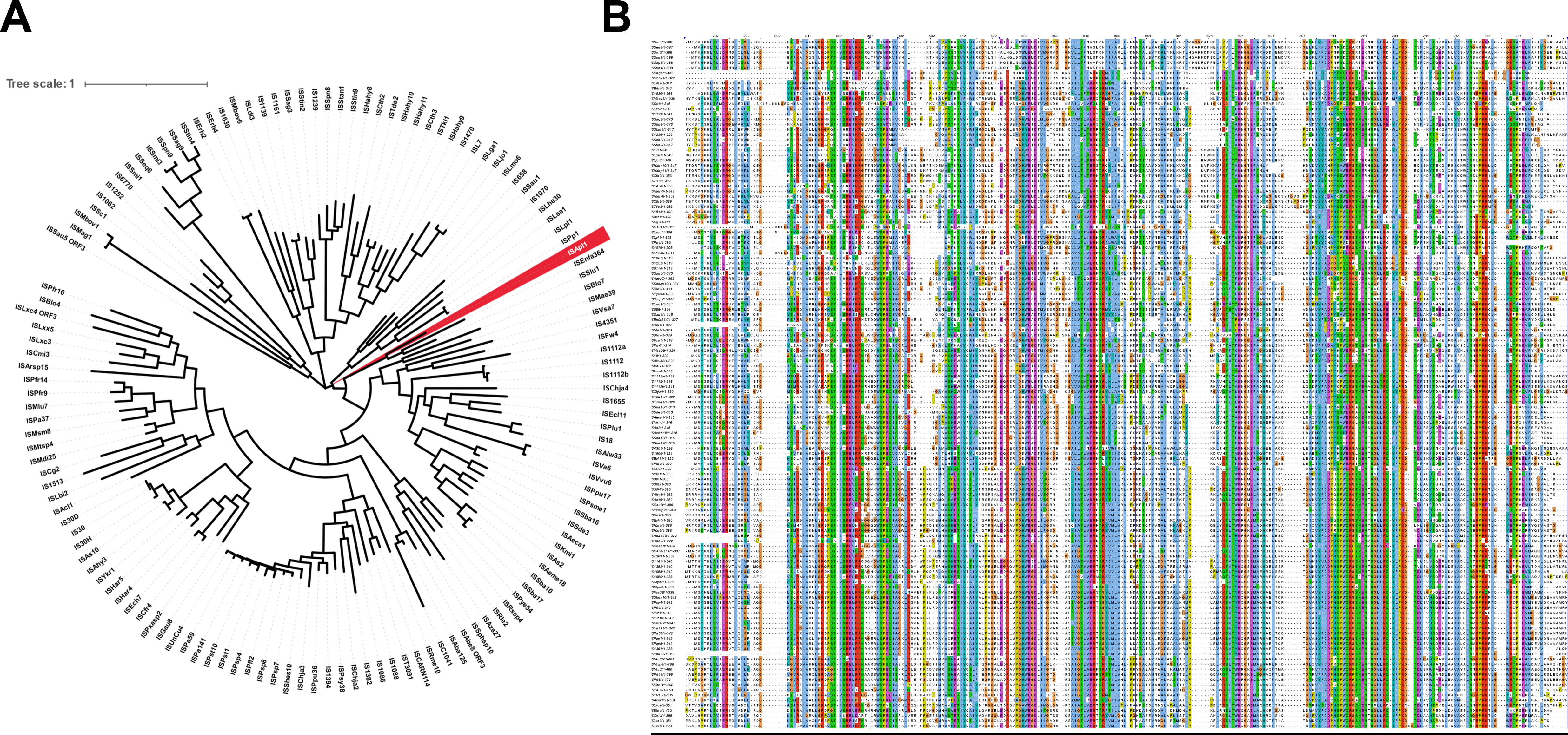

Supplement: Supplemental Fig S2 — IS30 family evolutionary tree and IS30-family-align. [file aac.01231-23-s0005.tif]

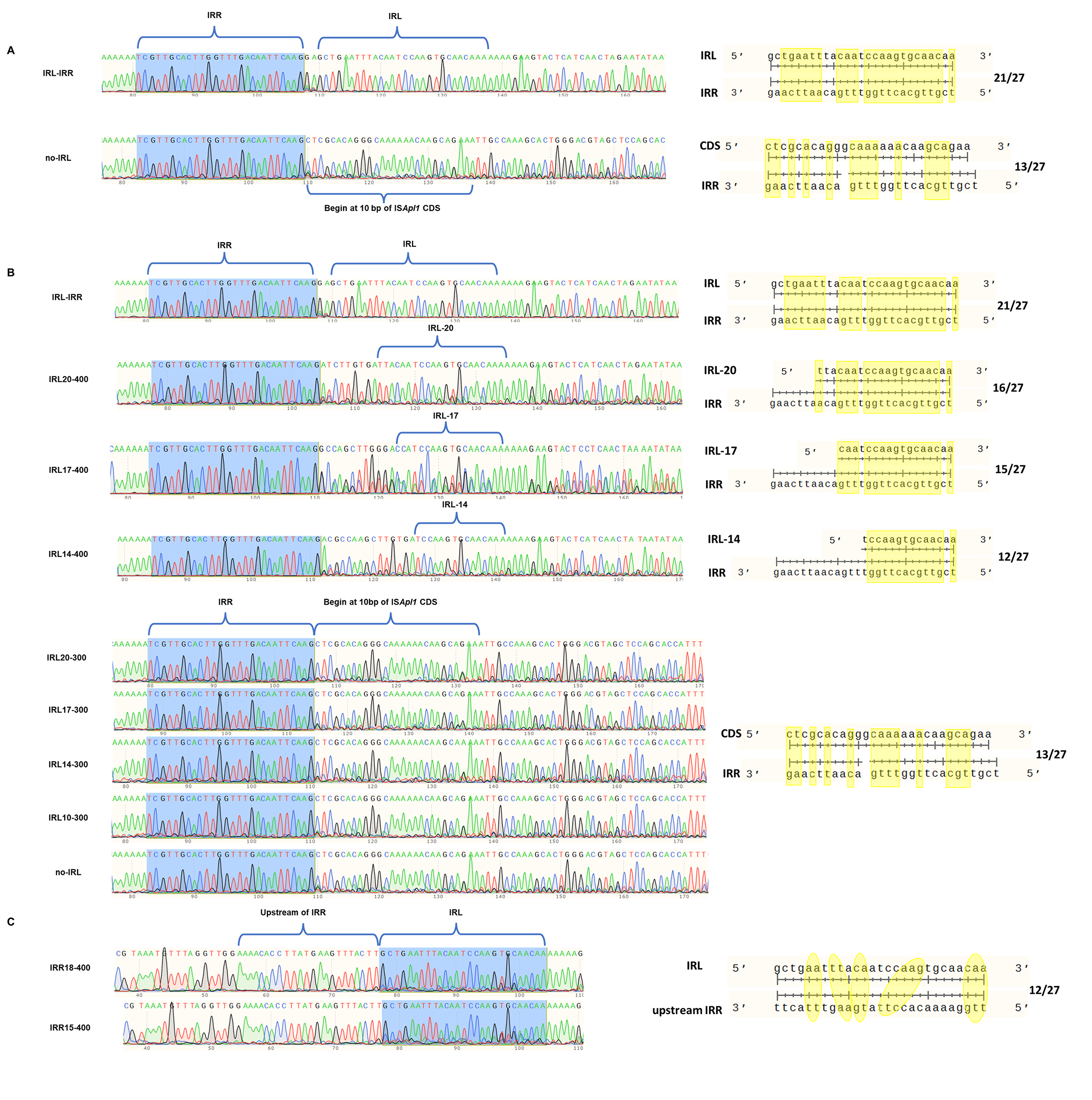

Supplement: Supplemental Fig S3 — Sanger sequence of cyclization product. [file aac.01231-23-s0006.tif]

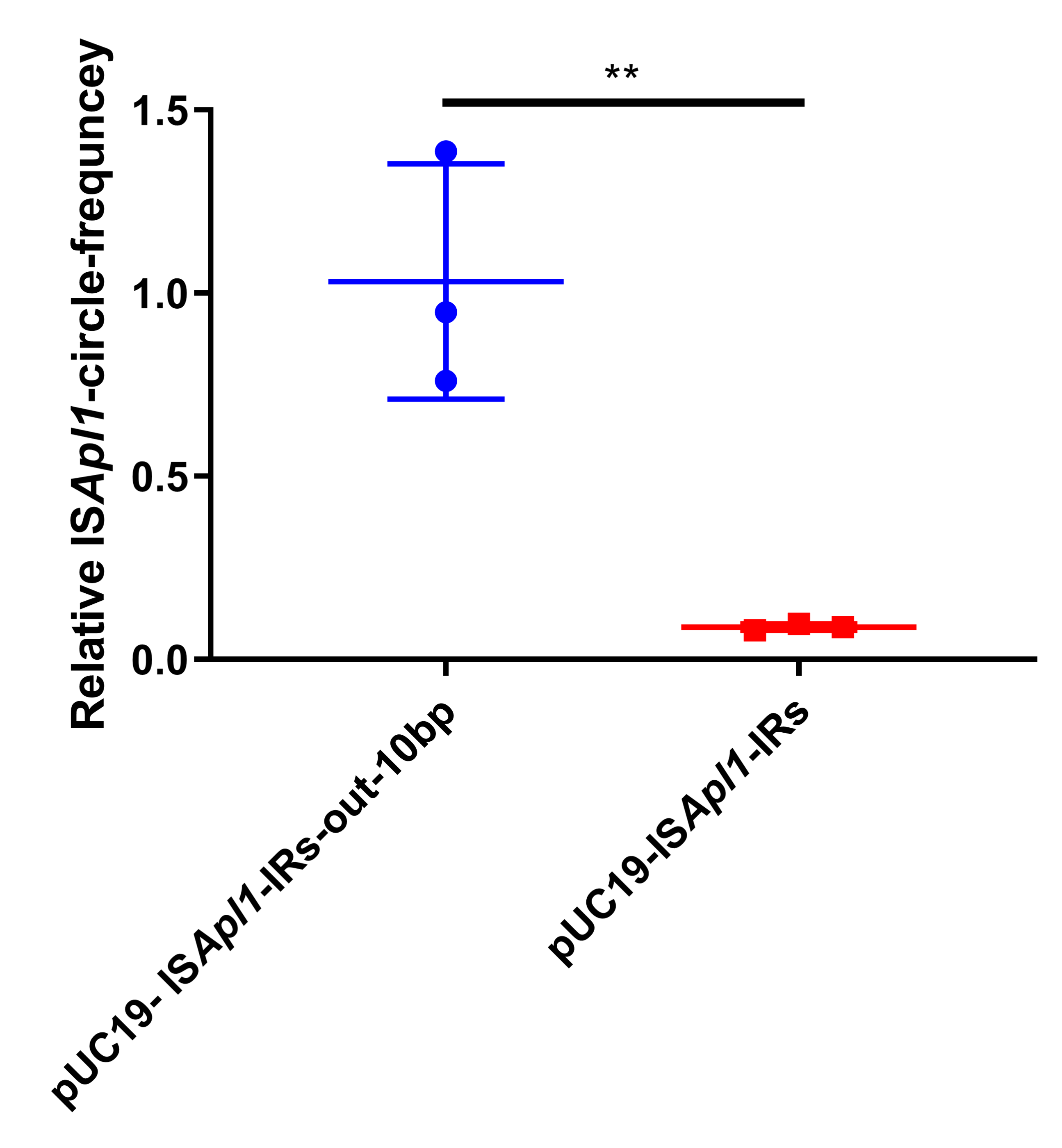

Supplement: Supplemental Fig S4 — Detection of cyclization frequency. [file aac.01231-23-s0007.tif]

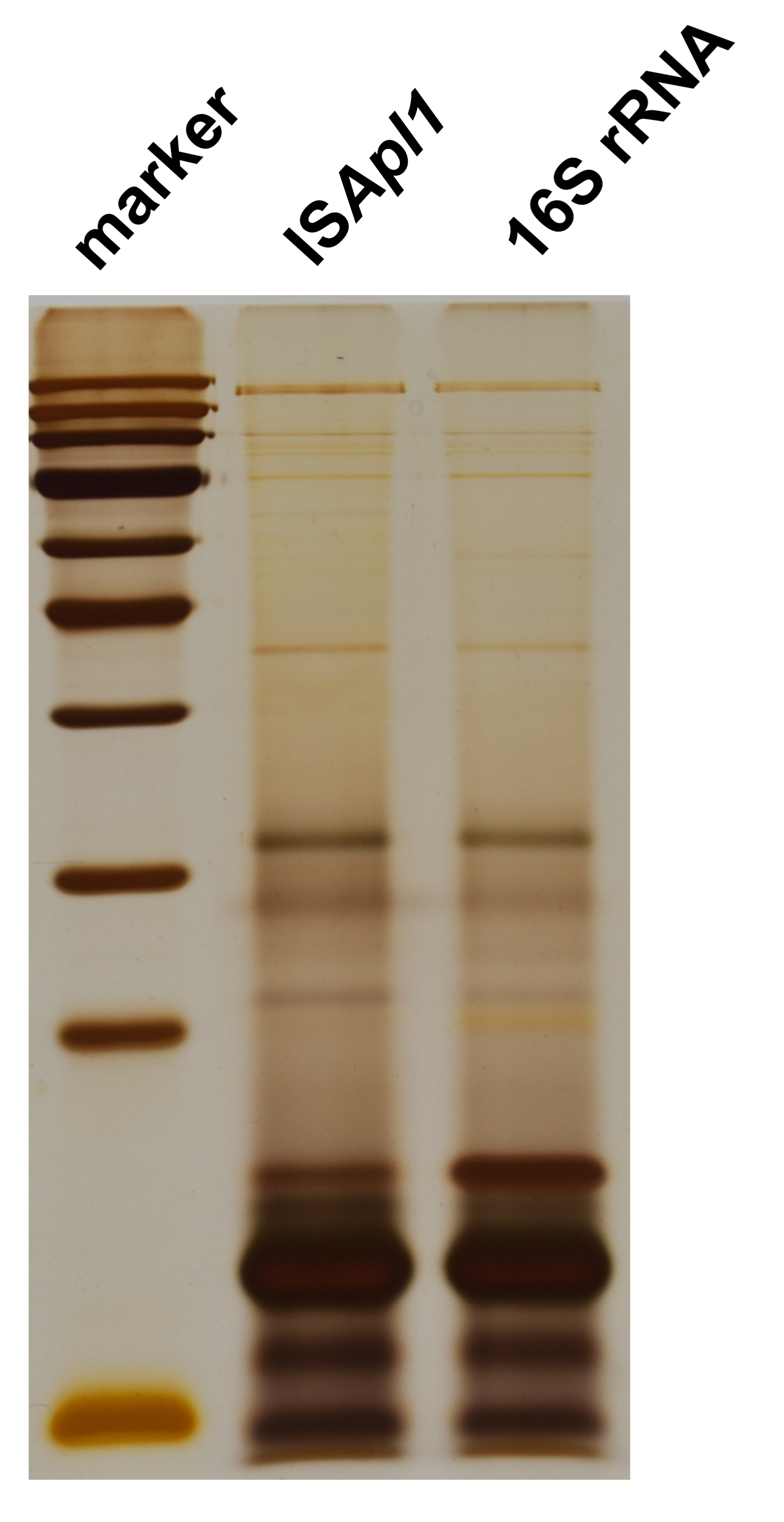

Supplement: Supplemental Fig S5 — Pull-down. [file aac.01231-23-s0008.tif]

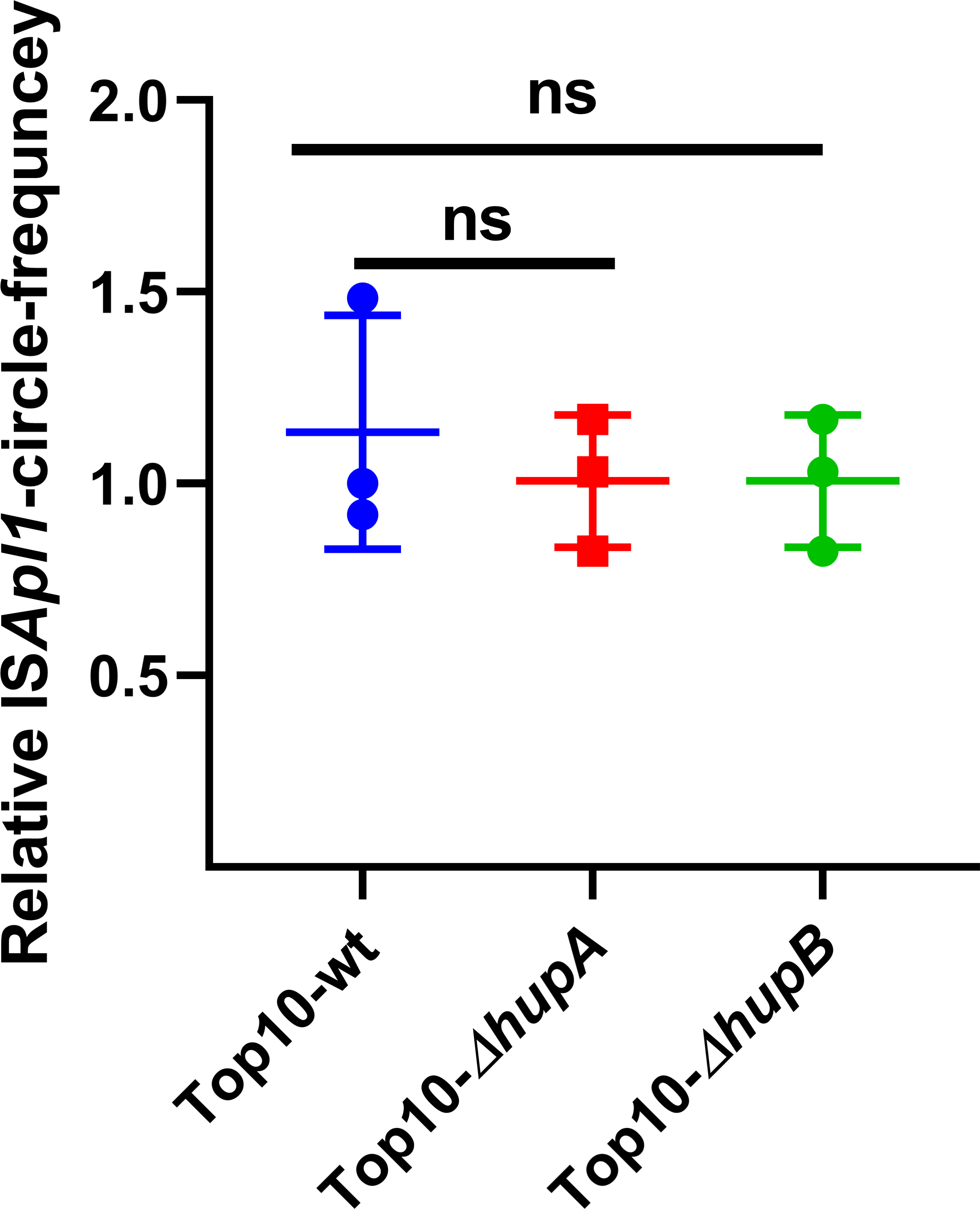

Supplement: Supplemental Fig S6 — Detection of cyclization frequency. [file aac.01231-23-s0009.tif]
