# Supplementary material for: Transposition mechanism of ISApl1—the determinant of colistin resistance dissemination
Source: Antimicrob Agents Chemother. 2024 Jan 30;68(3):e01231-23. doi: 10.1128/aac.01231-23 (PMC10916398; doi:10.1128/aac.01231-23)
Supplement: Supplemental Fig. S1-S7 and Tables S1-S2 — Fig. S1-S7 and Excel S1-S3 descriptions and lists of isolates, plasmids, and primers. [file aac.01231-23-s0010.docx]

Figure S1. Transposition of *mcr-1* depends on IS*Apl1* at both flanking ends.

(A) pSTV28 derivate plasmids Tn*6330*, ISMP, MPIS, and MP were constructed. Tn*6330* (IS*Apl1*-*mcr-1*-*pap2*-IS*Apl1*); ISMP (IS*Apl1*-*mcr-1*-*pap2*); MPIS (*mcr-1*-*pap2*-IS*Apl1*); MP (*mcr-1*-*pap2*). Verification using P3/P4 shows that transposition can only be completed if both flanking ends of *mcr-1* have IS*Apl1*.

(B) Five clinical isolates GBGD22 (MP), GBGD28 (ISMP), GBGD32 (MP), GBGD45 (MP), GBGD52 (Tn*6330*) were also detected. The verification using P3/P4 shows that transposition can only be completed if both flanking ends of *mcr-1* have IS*Apl1*.

Figure S2. Phylogenetic tree and the alignment of protein sequences of the IS*30* family.

(A) We downloaded the IS*30* family protein sequence and constructed a phylogenetic tree using MAGE software. IS*Apl1* is a marker in red.

(B) Using Clustal Omega, 134 transposases were aligned. Jalview was used to edit and beautify the sequences. In this figure we have hidden some non-conserved sequences.

Figure S3. Sanger sequence of the products.

(A) Sanger sequence of the cyclization products. The product sizes of pUC19-ISApl1-IRL-IRR (IRL-IRR) and pUC19-ISApl1-no-IRL (no-IRL) were 400 bp and 252 bp, respectively. The sequences for homologous recombination were IRL and CDS regions starting at 10 bp of ISApl1. IRL-IRR: products with 6 bp mismatches. No-IRL: products with 14 bp mismatches. Yellow indicates that the base pair can be matched. The light blue part is the IRR sequence.

(B) Sequence of the shortened IRL products. Shortening the length from whole-length IRL to 10 bp or no-IRL. Two sizes of products, approximately 400 bp and 252 bp, were sequenced. IRL-20-400: products with 11 bp mismatches. IRL-17-400: products with 12 bp mismatches. IRL-14-400: products with 15 bp mismatches. IRL-20-300: products with 14 bp mismatches. IRL-17-300: products with 14 bp mismatches. IRL-14-300: products with 14 bp mismatches. IRL-10-300: products with 14 bp mismatches. Yellow indicates that the base pair can be matched. The light blue part is the IRR sequence.

(C) Sequence of the shortened IRR products. Shortening the length from whole-length IRR to 15 bp. Products of approximately 400 bp were sequenced. IRL-18-400: products with 15 bp mismatches. IRL-15-400: products with 15 bp mismatches. Yellow indicates that the base pair can be matched. The light blue part is the IRR sequence.

Figure S4. Detection of IS*Apl1*-self-cyclization.

Two groups of pUC19 derivatives, with and without the original flanking 10 bp outside of IR. RT-qPCR detected the excision frequency. Student's two-tailed unpaired *t*-test was utilized to calculate significant differences, **P* < 0.05; ***P* < 0.01.

Figure S5. SDS-PAGE analysis of proteins binding to IS*Apl1* promoter region. The gel was treated with silver staining, and the whole band was treated by mass spectrometry. *16S rRNA* was a negative control.

Figure S6. Detection of IS*Apl1*-self-cyclization in mutant strains. Detect the excision of IS*Apl1* in *∆hupA* or *∆hupB* mutant strains. Student's two-tailed unpaired *t*-test was utilized to calculate significant differences. NS, not significant (*P* > 0.05).

Excel S1. The list of *mcr-1*-positive strains. (A) The antibacterial spectrum of *mcr-1* positive strains. (B) The location of *mcr-1*-positive stains. The distribution of strains from environmental and clinical sources in each province is detailed. (C) Type of plasmid carrying the *mcr-1* gene. The type of plasmid carrying the *mcr-1* gene and whether the plasmid contained the IS*Apl1* transposase gene were counted.

Excel S2. Pull-down mass spectrometry results of the IS*Apl1* probe.

Excel S3. Pull-down mass spectrometry results of the *16S rRNA* probe.

**Table S1** Bacterial strains used in this study

| **Strains or plasmids** | **Description** | **Source or reference** |
| --- | --- | --- |
| **Strains** |  |  |
| 17MR471 | Strain With Tn6330, NCBI: NZ_CP051158.1 | This study |
| 17MR471-pUCK19-IS*Apl1* | Overexpression IS*Apl1* in 17MR471 | This study |
| GBGD22 | Strain with *mcr-1-pap2* (MP), NCBI: NZ_RQTO00000000.1 | From Tian lab |
| GBGD32 | Strain with *mcr-1-pap2* (MP), NCBI: NZ_RQTY00000000.1 | From Tian lab |
| GBGD52 | Strain with Tn6330, NCBI: NZ_RQUS00000000.1 | From Tian lab |
| GBGD28 | Strain with IS*Apl1*-*mcr-1-pap2* (ISMP), NCBI: NZ_RQTU00000000.1 | From Tian lab |
| GBGD45 | Strain with *mcr-1-pap2* (MP), NCBI: NZ_RQUL00000000.1 | From Tian lab |
| BL21(DE3) | Expression strain | TransGen |
| Top10 | *F- mcrA Δ* (*mrr-hsdRMS-mcrBC) φ80 lacZΔM15 ΔlacX74 recA1 araΔ139 Δ* (*ara-leu)7697 galU galK rpsL* (*StrR) endA1 nupG* | Shang Hai Wei Di |
| Top10 (pSTV28+Tn6330) | pSTV28 with Tn6330 | This study |
| Top10 (pSTV28+ISMP) | pSTV28 with ISMP | This study |
| Top10 (pSTV28+MPIS) | pSTV28 with MPIS | This study |
| Top10 (pSTV28+MP) | pSTV28 with MP | This study |
| Top10 (pUC19-IS*Apl1*-IRs) | IS*Apl1* with IRL and IRR | This study |
| Top10 (pUC19-IS*Apl1*-no-IR) | IS*Apl1* without IRL and IRR | This study |
| Top10 (pUC19+ IS*Apl1*-no-IRR) | IS*Apl1* without IRR | This study |
| Top10 (pUC19+ IS*Apl1*-no-IRL) | IS*Apl1* without IRL | This study |
| Top10 (pUC19+WT-IS*Apl1*-WT) | IS*Apl1* with IRL and IRR and including both sides of out 10bp | This study |
| Top10 (pUC19+WT-IS*Apl1*-W) | IS*Apl1* with IRL (including out 10bp) and IRR (including random 10bp W) | This study |
| Top10 (pUC19+WT-IS*Apl1*-S) | IS*Apl1* with IRL (including out 10bp) and IRR (including random 10bp S) | This study |
| Top10 (pUC19+W-IS*Apl1*-WT) | IS*Apl1* with IRL (including random 10bp W 10bp) and IRR (including out 10bp) | This study |
| Top10 (pUC19+W-IS*Apl1*-W) | IS*Apl1* with IRL (including random 10bp W) and IRR (including random 10bp W) | This study |
| Top10 (pUC19+S-IS*Apl1*-WT) | IS*Apl1* with IRL (including random 10bp W) and IRR (including random 10bp WT) | This study |
| Top10 (pUC19+S-IS*Apl1*-S) | IS*Apl1* with IRL (including random 10bp S) and IRR (including random 10bp S) | This study |
| Top10 (pUC19+IRL (20bp)-IS*Apl1*-IRR) | IS*Apl1* with IRL (20bp) and IRR (27bp) | This study |
| Top10 (pUC19+IRL (17bp)-IS*Apl1*-IRR) | IS*Apl1* with IRL (17bp) and IRR (27bp) | This study |
| Top10 (pUC19+IRL (14bp)-IS*Apl1*-IRR) | IS*Apl1* with IRL (14bp) and IRR (27bp) | This study |
| Top10 (pUC19+IRL (13bp)-IS*Apl1*-IRR) | IS*Apl1* with IRL (13bp) and IRR (27bp) | This study |
| Top10 (pUC19+IRL (12bp)-IS*Apl1*-IRR) | IS*Apl1* with IRL (12bp) and IRR (27bp) | This study |
| Top10 (pUC19+IRL (11bp)-IS*Apl1*-IRR) | IS*Apl1* with IRL (11bp) and IRR (27bp) | This study |
| Top10 (pUC19+IRL (10bp)-IS*Apl1*-IRR) | IS*Apl1* with IRL (10bp) and IRR (27bp) | This study |
| Top10 (pUC19+IRL-IS*Apl1*-IRR (18bp)) | IS*Apl1* with IRL (27bp) and IRR (18bp) | This study |
| Top10 (pUC19+IRL-IS*Apl1*-IRR (15bp)) | IS*Apl1* with IRL (27bp) and IRR (15bp) | This study |
| Top10 (pUC19+IRL-IS*Apl1*-IRR (12bp)) | IS*Apl1* with IRL (27bp) and IRR (12bp) | This study |
| Top10 (pUC19+IRL-IS*Apl1*-IRR (11bp)) | IS*Apl1* with IRL (27bp) and IRR (11bp) | This study |
| Top10 (pUC19+IRL-IS*Apl1*-IRR (10bp)) | IS*Apl1* with IRL (27bp) and IRR (10bp) | This study |
| Top10 (pUC19+IRL-IS*Apl1*-IRR (9bp)) | IS*Apl1* with IRL (27bp) and IRR (9bp) | This study |
| Top10 (pUC19+IS*Apl1*-kan) | Kanamycin resistance gene insert IS*Apl1* | This study |
| Top10 (pUC19+IS*Apl1*-kan+pSTV28-IS*Apl1*) | In the Top10 (pUC19+IS*Apl1*-kan) complement IS*Apl1* | This study |
| Top10 (pUC19+IS*Apl1*-kan+pSTV28-IS*Apl1*-D163A) | In the Top10 (pUC19+IS*Apl1*-kan) complement IS*Apl1* mutant D163A | This study |
| Top10 (pUC19+IS*Apl1*-kan+pSTV28-IS*Apl1*-D217A) | In the Top10 (pUC19+IS*Apl1*-kan) complement IS*Apl1* mutant D217A | This study |
| Top10 (pUC19+IS*Apl1*-kan+pSTV28-IS*Apl1*-E251A) | In the Top10 (pUC19+IS*Apl1*-kan) complement IS*Apl1* mutant E251A | This study |
| Top10-∆*hupA* | Top10 *hupA* mutant strain | This study |
| Top10-∆*hupB* | Top10 *hupB* mutant strain | This study |
| **Plasmids** |  |  |
| pSTV28 | Complement plasmid, cm^R^ | From Xue lab |
| pUC19 | Target plasmid used in strand transfer assays, Amp^R^ | Shang Hai Wei Di |
| pUCK19 | pUC19 with kan resistance gene | This study |
| pUCK19-IS*Apl1* | Overexpression IS*Apl1* | This study |
| pUCK19-*hupA* | Overexpression *hupA* | This study |
| pUCK19-*hupB* | Overexpression *hupB* | This study |
| pET28(+) | Expression vector with a hexa-histidine tag, Kan^r^ | Novagen |
| pKD46 | Expresses λ Red recombinase Exo, Bet and Gam,  temperature sensitive, Ampr | From Xue lab |
| pKD3 | cat gene, template plasmid, Ampr Cm r | From Xue lab |
| pCP20 | FLP+ λc I857+ λpRRep (Ts), temperature sensitive, Ampr Cmr | From Xue lab |
|  |  |  |
|  |  |  |

cm^R^, resistance to chloramphenicol; Amp^R^, resistance to ampicillin.

**Table S2** Primers used in this study

| **Primer name** | **Primer sequence (5′to 3′)** | **Description** |
| --- | --- | --- |
| P1: IS*Apl1*-R | gtgcgttcgttgcactgtag | Detect cyclization |
| P2: IS*Apl1*-F | gacatcaatcagtggagcgaagtt | Detect cyclization |
| P3: *mcr-1*-R | gctgcatcatgagaaactactc | Detect cyclization |
| P4: *pap2-R* | ggcgatgggctgctataaga | Detect cyclization |
| IS*Apl1*-F (*Eco*R I)-pSTV28 | GGAATTCC atgttggcgaatccacagttcaacccagaatagcgcagt | Construct plasmid |
| *mcr-1*-F (*Eco*R I)- pSTV28 | GGAATTCC atacaaattataaatactctcaagtgtatattcagtatggg | Construct plasmid |
| IS*Apl1*-R (*Sal* I)- pSTV28 | ACGCGTCGAC catccgacatccggagtaccatgcctgatacgacgctt | Construct plasmid |
| *pap2*-R (*Sal* I)-pSTV28 | ACGCGTCGAC cgattaaacttgttcacccttcttaaaaaacacccacgcaag | Construct plasmid |
| IS*Apl1*-F+IRL (*Hind* III)-out (10bp) | CCAAGCTTGTGA agtttaatcggctgaatttacaatccaagtgcaacaa | Construct plasmid |
| IS*Apl1*-R-IRR (*Sal* I)-out (10bp) | GCGTCGAC aaatattacccttgaattgtcaaaccaagtgcaacga | Construct plasmid |
| IS*Apl1*-F+IRL (*Hind* III) | CCAAGCTTGTGA gctgaatttacaatccaagtgcaacaa | Construct plasmid |
| IS*Apl1*-R-IRR (*Sal* I) | GCGTCGAC cttgaattgtcaaaccaagtgcaacga | Construct plasmid |
| IS*Apl1*-F+IRL (*Hind* III)-NO-IRL | CCAAGCTTGTGA aaaaagaagtactcatcaactagaat | Construct plasmid |
| IS*Apl1*-R-IRR (*Sal* I)-NO-IRR | GCGTCGAC tcattttttgaagtaaacttcat | Construct plasmid |
| IS*Apl1*-F+IRL (*Hind* III)-out (10bp-S) | CCAAGCTTGTGA ssssssssss gctgaatttacaatccaagtgcaacaa | Construct plasmid |
| IS*Apl1*-F+IRL (*Hind* III)-out (10bp-W) | CCAAGCTTGTGAwwwwwwwwww  gctgaatttacaatccaagtgcaacaa | Construct plasmid |
| IS*Apl1*-R-IRR (*Sal* I)-out (10bp-S) | GCGTCGAC sssssssssscttgaattgtcaaaccaagt | Construct plasmid |
| IS*Apl1*-R-IRR (*Sal* I)-out (10bp-W) | GCGTCGAC wwwwwwwwwwcttgaattgtcaaaccaagt | Construct plasmid |
| IS*Apl1*-R-IRR-18 (*Sal* I) | GCGTCGAC tcaaaccaagtgcaacgatt | Construct plasmid |
| IS*Apl1*-R-IRR-15 (*Sal* I) | GCGTCGAC tcaaaccaagtgcaacgattttt | Construct plasmid |
| IS*Apl1*-R-IRR-12 (*Sal* I) | GCGTCGAC tcacaagtgcaacgattttttga | Construct plasmid |
| IS*Apl1*-R-IRR-11 (*Sal I*) | GCGTCGAC tcaaagtgcaacgattttttgaagt | Construct plasmid |
| IS*Apl1*-R-IRR-10 (*Sal I*) | GCGTCGAC tcaagtgcaacgattttttgaagta | Construct plasmid |
| IS*Apl1*-R-IRR-9 (*Sal I*) | GCGTCGAC tcagtgcaacgattttttgaagt | Construct plasmid |
| IS*Apl1*-F-IRL-23 (*Hind* III) | CCAAGCTTGTGA aatttacaatccaagtgcaa | Construct plasmid |
| IS*Apl1*-F-IRL-20 (*Hind* III) | CCAAGCTTGTGA ttacaatccaagtgcaacaa | Construct plasmid |
| IS*Apl1*-F-IRL-17 (*Hind* III) | CCAAGCTTGTGA caatccaagtgcaacaaaaa | Construct plasmid |
| IS*Apl1*-F-IRL-14 (*Hind* III) | CCAAGCTTGTGA tccaagtgcaacaaaaaaagaa | Construct plasmid |
| IS*Apl1*-F-IRL-13 (*Hind* III) | CCAAGCTTGTGA ccaagtgcaacaaaaaaagaag | Construct plasmid |
| IS*Apl1*-F-IRL-12 (*Hind* III) | CCAAGCTTGTGA caagtgcaacaaaaaaagaagt | Construct plasmid |
| IS*Apl1*-F-IRL-11 (*Hind* III) | CCAAGCTTGTGA aagtgcaacaaaaaaagaag | Construct plasmid |
| IS*Apl1*-F-IRL-10 (*Hind* III) | CCAAGCTTGTGA agtgcaacaaaaaaagaagt | Construct plasmid |
| IS*Apl1*-F- (*Eco*R I)- pSTV28-C | CGGAATTC aaaaagaagtactcatcaactagaat | Construct plasmid |
| IS*Apl1*-R (*Sal* I)-pSTV28-C | GCGTCGAC tcaaaccaagtgcaacgatt | Construct plasmid |
| IS*Apl1*-1-F (*Hind* III) | CCAAGCTTGTGAcctggcgggcttttttatgc | Construct plasmid |
| IS*Apl1*-1-R (*Sal I*) | GCGTCGACctatctattaaatcatcttgaattg | Construct plasmid |
| IS*Apl1*-2-F (*Hind* III) | CCAAGCTTGTGAagggattgtttttcgaggct | Construct plasmid |
| IS*Apl1*-2-R (*Sal I*) | GCGTCGACtgactactgtttttatctctt | Construct plasmid |
| IS*Apl1*-3-F (*Hind* III) | CCAAGCTTGTGAagtgccatgtttttattggc | Construct plasmid |
| IS*Apl1*-3-R (*Sal I*) | GCGTCGACgggctttaaacacttgaatt | Construct plasmid |
| IS*Apl1*-4-F (*Hind* III) | CCAAGCTTGTGAgatatttcattggggctga | Construct plasmid |
| IS*Apl1*-4-R (*Sal I*) | GCGTCGACtatttataatttgtatcttgaa | Construct plasmid |
| IS*Apl1*-5-F (*Hind* III) | CCAAGCTTGTGAgtttaatcggctgaatttaca | Construct plasmid |
| IS*Apl1*-5-R (*Sal I*) | GCGTCGACaccaaaatattacccttgaat | Construct plasmid |
| IS*Apl1*-6-F (*Hind* III) | CCAAGCTTGTGAtgatattttgagctgaatttac | Construct plasmid |
| IS*Apl1*-6-R (*Sal I*) | GCGTCGACtatttataatttgtatcttgaa | Construct plasmid |
| IS*Apl1*-7-F (*Hind* III) | CCAAGCTTGTGAgtttaatcggctgaatttaca | Construct plasmid |
| IS*Apl1*-7-R (*Sal I*) | GCGTCGACatgtggtcggagatccttg | Construct plasmid |
| IS*Apl1*-8-F (*Hind* III) | CCAAGCTTGTGAggtctctttttatctgctgaa | Construct plasmid |
| IS*Apl1*-8-R (*Sal I*) | GCGTCGACtctggcttttaacagcttga | Construct plasmid |
| IS*Apl1*-9-F (*Hind* III) | CCAAGCTTGTGAcgcattgttttttttatgctga | Construct plasmid |
| IS*Apl1*-9-R (*Sal I*) | GCGTCGACaataaatatatcttgaattgtcaaac | Construct plasmid |
| IS*Apl1*-10-F (*Hind* III) | CCAAGCTTGTGAcatttttacagagctgaatt | Construct plasmid |
| IS*Apl1*-10-R (*Sal I*) | GCGTCGACggatcgattattaatccttga | Construct plasmid |
| IS*Apl1*-11-F (*Hind* III) | CCAAGCTTGTGAcagattttttttgatgctgaa | Construct plasmid |
| IS*Apl1*-11-R (*Sal I*) | GCGTCGACggaactattagtaaatcttg | Construct plasmid |
| IS*Apl1*-12-F (*Hind* III) | CCAAGCTTGTGAcgatttttattgcgctgaat | Construct plasmid |
| IS*Apl1*-12-R (*Sal I*) | GCGTCGACcaaatctttagccttgaattg | Construct plasmid |
| *kan*-F-overlap | gttgtttgatataggcgaacgcatctcaagaagatcctttg | Amplification of kan resistance gene |
| *kan*-R-overlap | cgcagtttgcgactggctttttagaaaaactcatcgagcatc | Amplification of kan resistance gene |
| DDE-D163A-F | ttgggaagccgccaccgtac | Construct point mutation |
| DDE-D163A-R | gtacggtggcggcttcccaa | Construct point mutation |
| DDE-D217A-F | aattacgccagcccgtggta | Construct point mutation |
| DDE-D217A-R | taccacgggctggcgtaatt | Construct point mutation |
| DDE-E251A-F | ggggaacgaatgccaataca | Construct point mutation |
| DDE-E251A-R | tgtattggcattcgttcccc | Construct point mutation |
| IS*Apl1*-F-probe | ggcagtcgttgatagtggatgg | For EMSA |
| IS*Apl1*-F-IRL-27 | gctgaatttacaatccaagtgcaacaa | For EMSA |
| IS*Apl1*-F-IRL-23 | aatttacaatccaagtgcaa | For EMSA |
| IS*Apl1*-F-IRL-20 | ttacaatccaagtgcaacaa | For EMSA |
| IS*Apl1*-F-IRL-17 | caatccaagtgcaacaaaaa | For EMSA |
| IS*Apl1*-F-IRL-14 | tccaagtgcaacaaaaaaagaa | For EMSA |
| IS*Apl1*-F-IRL-13 | ccaagtgcaacaaaaaaagaag | For EMSA |
| IS*Apl1*-F-IRL-12 | caagtgcaacaaaaaaagaagt | For EMSA |
| IS*Apl1*-F-IRL-11 | aagtgcaacaaaaaaagaag | For EMSA |
| IS*Apl1*-F-IRL-10 | agtgcaacaaaaaaagaagt | For EMSA |
| IS*Apl1*-F-NO-IRL | aaaaagaagtactcatcaactagaat | For EMSA |
| *mcr-1*-R-probe | cccatactgaatatacacttgagag | For EMSA |
| IS*Apl1*-R-IRR-27 | cttgaattgtcaaaccaagt | For EMSA |
| IS*Apl1*-R-IRR-18 | tcaaaccaagtgcaacgatt | For EMSA |
| IS*Apl1*-R-IRR-15 | aaccaagtgcaacgattttt | For EMSA |
| IS*Apl1*-R-IRR-12 | caagtgcaacgattttttga | For EMSA |
| IS*Apl1*-R-IRR-11 | aagtgcaacgattttttgaagt | For EMSA |
| IS*Apl1*-R-IRR-10 | agtgcaacgattttttgaagta | For EMSA |
| IS*Apl1*-R-IRR-9 | gtgcaacgattttttgaagt | For EMSA |
| IS*Apl1*-R-NO-IRR | ttttttgaagtaaacttcat | For EMSA |
| IS*Apl1*-F-IRL-10WT | agtttaatcggctgaatttacaatccaagtgcaacaa | For EMSA |
| IS*Apl1*-F-IRL-10S | cccgcgccgcgctgaatttacaatccaagtgca | For EMSA |
| IRR-R-10WT | aaatattacccttgaattgtcaaaccaagtgc | For EMSA |
| IRR-R-10S | cccgcgccgcgccttgaattgtcaaaccaagtgc | For EMSA |
| IS*Apl1*-R-biotin | gccctgtgcgagtaaaatc | For Pull Down |
| IS*Apl1*-F-pull-down | ggcagtcgttgatagtggatgg | For Pull Down and EMSA |
| IS*Apl1*-F-FAM | aaactgaatttacgaccacg | Labeled with 6-FAM |
| IS*Apl1*-R-FAM | gccctgtgcgagtaaaatc | Labeled with 6-FAM |
| IS*Apl1*-F- *Hind* III | GATTACGCCAAGCTTGTAA aaaaagaagtactcatcaactag | Overexpression IS*Apl1* |
| IS*Apl1*-R- *Sal* I | CGGGGATCCTCTAGAGTCGAC tcaaaccaagtgcaacgatt | Overexpression IS*Apl1* |
| *hupA*-F- *Hind* III | CCAAGCTTtcagcaataagaccagaa | Overexpression *hupA* |
| *hupA*-R- *Sal* I | GCGTCGACctgccacgcaatcttactta | Overexpression *hupA* |
| *hupB*-F- *Hind* III | CCAAGCTTtgcaaaatagtgacctcgc | Overexpression *hupB* |
| *hupB*-R- *Sal* I | GCGTCGACggacaacgcttagtttac | Overexpression *hupB* |
| IS*Apl1*-F - *Sac* I | CGAGCTCatgatgagtacttcctaccg | Expression protein HTH |
| IS*Apl1*-R - *Xho* I | CCCTCGAGatattcaggctgctctaatt | Expression protein HTH |
| *hupA*-F- *BamH* I | CGCGGATCCGCGcttatgaacaagactcaactgattg | Expression protein *hupA* |
| *hupA*-R- *EcoR* I | CCGGAATTCCGGctgccacgcaatcttactta | Expression protein *hupA* |
| *hupB*-F- *BamH* I | CGCGGATCCGCGagaagagtgaataaatctc | Expression protein *hupB* |
| *hupB*-R- *EcoR* I | CCGGAATTCCGGggacaacgcttagtttac | Expression protein *hupB* |
| *hupA*-Δf | cgataaacacattgtaaggataacttatgaacaagactcatgtaggctggagctgctt | Knockout *hupA* |
| *hupA*-Δr | ttcgttaaaactgttcactgccacgcaatcttacttaactcatatgaatatcctcctta | Knockout *hupA* |
| *hupB*-Δf | gcgatataaattataaagaggaagagaagagtgaataaattgtaggctggagctgctt | Knockout *hupB* |
| *hupB*-Δr | gacaacgcttagtttaccgcgtctttcagtgctttacctgcatatgaatatcctcctta | Knockout *hupB* |
| pUC19-RT-F | cgttatcccctgattctgtg | RT-QPCR |
| pUC19-RT-R | tttgcgtattgggcgctctt | RT-QPCR |
| IS*Apl1*-circle-F | aaactgaatttacgaccacg | RT-QPCR |
| IS*Apl1*-circle-R | tttggaaatggtttttgtgtgg | RT-QPCR |
| 16srRNA-RT-F | cgaggtcgcttctctttg | RT-QPCR |
| 16srRNA-RT-R | ggagactgccagtgataaac | RT-QPCR |
| IS*Apl1*-RT-F | ctacagtgcaacgaacgc | RT-QPCR |
| IS*Apl1*-RT-R | aatcgtgcgctgatttgt | RT-QPCR |

Capital letters: restriction endonuclease recognition sites.

w: A, T; s: C, G
